# DNA Methylation Dynamics in Human Induced Pluripotent Stem Cells over Time

**DOI:** 10.1371/journal.pgen.1002085

**Published:** 2011-05-26

**Authors:** Koichiro Nishino, Masashi Toyoda, Mayu Yamazaki-Inoue, Yoshihiro Fukawatase, Emi Chikazawa, Hironari Sakaguchi, Hidenori Akutsu, Akihiro Umezawa

**Affiliations:** Department of Reproductive Biology, National Institute for Child Health and Development, Tokyo, Japan; Albert Einstein College of Medicine, United States of America

## Abstract

Epigenetic reprogramming is a critical event in the generation of induced pluripotent stem cells (iPSCs). Here, we determined the DNA methylation profiles of 22 human iPSC lines derived from five different cell types (human endometrium, placental artery endothelium, amnion, fetal lung fibroblast, and menstrual blood cell) and five human embryonic stem cell (ESC) lines, and we followed the aberrant methylation sites in iPSCs for up to 42 weeks. The iPSCs exhibited distinct epigenetic differences from ESCs, which were caused by aberrant methylation at early passages. Multiple appearances and then disappearances of random aberrant methylation were detected throughout iPSC reprogramming. Continuous passaging of the iPSCs diminished the differences between iPSCs and ESCs, implying that iPSCs lose the characteristics inherited from the parent cells and adapt to very closely resemble ESCs over time. Human iPSCs were gradually reprogrammed through the “convergence” of aberrant hyper-methylation events that continuously appeared in a de novo manner. This iPS reprogramming consisted of stochastic de novo methylation and selection/fixation of methylation in an environment suitable for ESCs. Taken together, random methylation and convergence are driving forces for long-term reprogramming of iPSCs to ESCs.

## Author Summary

iPSCs change to resemble ESCs via two phases: the transgene-dependent phase, in which the transcription factors act to transform somatic cells into pluripotent stem cells, and the transgene-independent phase, in which the transcription factors are silenced. In this study, we established human iPSCs derived from 5 different cell types by retroviral infection of the Yamanaka 4 factors, and we identified 8 novel epigenetic markers (*SALL4*, *EPHA1*, *PTPN6*, *RAB25*, *GBP4*, *LYST*, *SP100*, and *UBE1L*) by comprehensive DNA methylation analysis. The aberrant hyper-methylation in iPSCs occurred stochastically throughout the genome and decreased during the long-term iPSC reprogramming, suggesting that the aberrant stochastic hyper-methylation and their convergence are a direct cause of the transgene-independent phase of iPS reprogramming. These results favor the stochastic model of the Yamanaka model rather than the elite model. In addition, the stem cell–specific methylation states and the epigenetic difference between iPSCs and ESCs are useful indices for evaluating human iPSCs in therapeutic applications.

## Introduction

DNA methylation is an important epigenetic modification and is a key component in normal differentiation, development and disease [1]–[3]. Expression of tissue-specific genes, such as *Oct-4* [4], *Nanog* [5], *Sry* (sex determining region on Y chromosome) [6] and *MyoD* [7], are induced by spatio-temporal demethylation during development. DNA methylation therefore specifically varies depending on tissue types and cell linage [2], indicating that information regarding cell type-specific DNA methylation profiles can enable the identification and validation of cell types. Transformation of iPSCs from somatic cells requires a process of epigenetic reprogramming promoted by transient ectopic expression of defined transcription factors expressed in ESCs [8]–[11]. Human iPSCs are considered to be powerful resources in regenerative medicine because of their potential of pluripotency and avoidance of rejection of their derivatives by the immune system, and for ethical issues as well [12]. Although iPSCs show pluripotency, they have different propensities for differentiation in mouse models [13]. Human iPSCs also exhibit donor cell-specific gene expression [14], [15]. Moreover, iPSCs possess inherited DNA methylation states as epigenetic memories from parent cells [15]–[17], suggesting that these memories influence different propensities of the iPSCs. On the other hand, continuous passaging of mouse iPSCs reduces differences from each other in gene expression profiles [15]. Epigenome-wide analysis started to be used in this field [18], [19], and differentially methylated regions have been identified among human iPSCs, their parent cells and ESCs [17], [20]. Aberrant epigenetic reprogramming has recently been reported in human iPSCs [21], [22]. However, these analyses were limited to the use of a small number of cells as a source for generation of iPS cells. Moreover, human iPSCs have only been analyzed at a single point of passage. Therefore, it has not been clarified whether human iPSCs generated from various types of cells are dissimilar from each other at different points during passage; how continuous passaging of human iPSCs influences the differences between iPSCs and ESCs; and how aberrant methylation in human iPSCs during passaging. To address these issues, we compared the epigenetic and transcriptional states of human iPSCs derived from five cell types of different origins during passage, and found random aberrant hyper-methylation at different points of adaptation into ESCs.

## Results

### Establishment of human iPSCs

**Figure 1:**
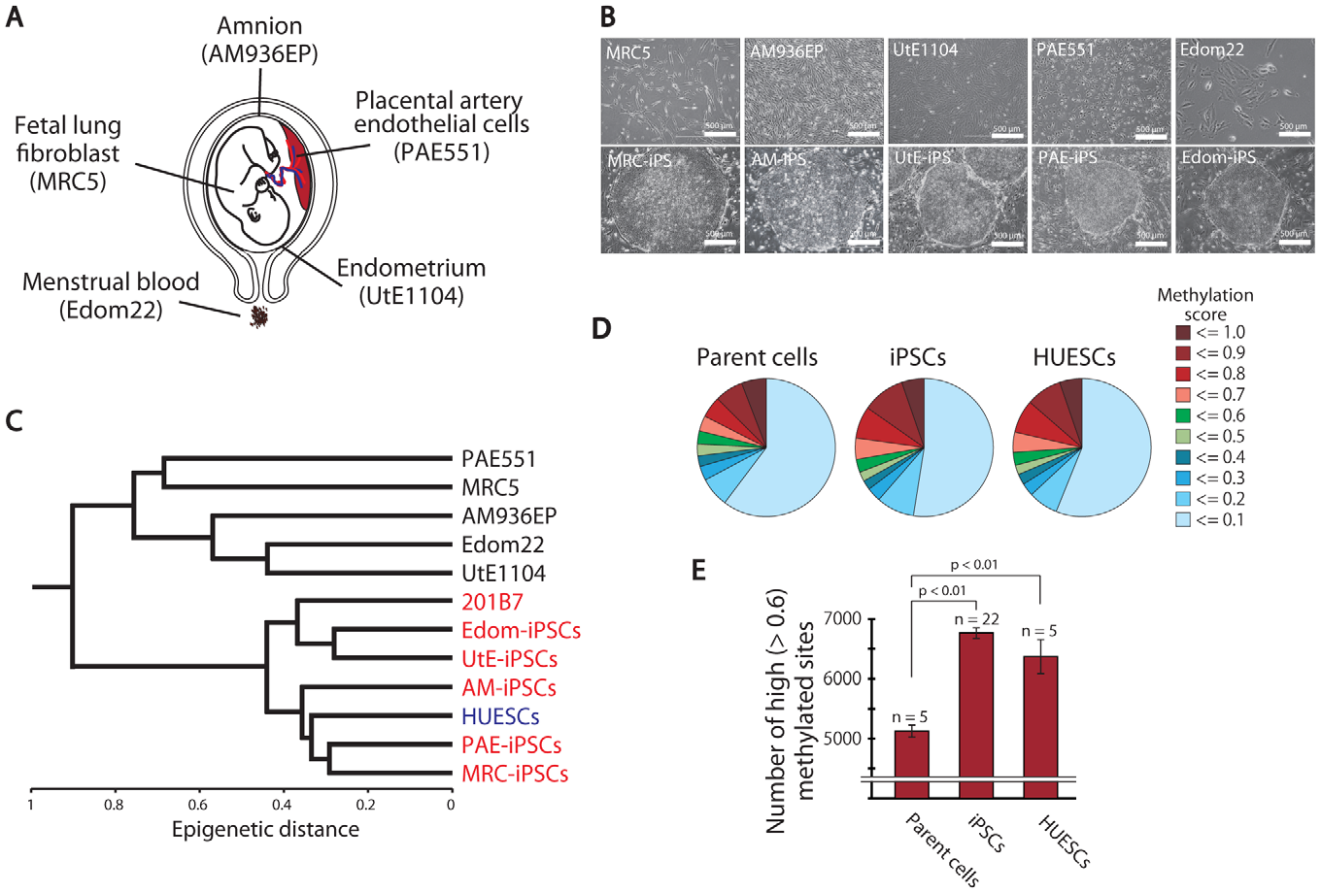
Pluripotent stem cells are significantly more hyper-methylated than their parent cells. (*A*) The human cell origins used for generation of iPSCs. (*B*) Morphology of the parent cells (upper panels) and iPSCs (lower panels). (*C*) Unsupervised hierarchical clustering analysis based on DNA methylation. (*D*) Distribution of 24,273 CpG sites with their methylation scores in the parent cells, iPSCs and ESCs. (*E*) The average number of high (>0.6) methylated CpG sites. The iPSCs have more highly methylated sites than the parent cells.

Human iPSCs derived from fetal lung fibroblasts (MRC5), amnion (AM), endometrium (UtE), placental artery endothelium (PAE) and menstrual blood cells (Edom) were independently established in our laboratory by retroviral infection of 4 genes (*OCT-3/4*, *SOX2*, *c-MYC*, and *KLF4*) (Figure 1A, 1B and Table S1). These cells clearly showed human ES-like characters in terms of morphology; cell-surface antigens; gene expression of stem cell markers; teratoma formation in which these cells differentiated to various tissues including neural tissues (ectoderm), cartilage (mesoderm), and epithelial tissues (endoderm); growth (more than 20 passages); and DNA methylation patterns at *OCT-3/4* and *NANOG* promoter regions (Figures S1, S2, S3). Short tandem repeat (STR) analysis showed clonality between the respective iPSC lines and their parent cells (Table S2). Silencing of transgenes and normal karyotypes of iPSCs were also confirmed (Figure S4 and Table S3).

### Analysis of DNA methylation profiles

To investigate the dynamics of DNA methylation in pluripotent stem cells, we examined 5 ESC lines (HUESCs) [23], [24], 22 iPSC lines, their parent cells and 201B7, using Illumina's Infinium HumanMethylation27 BeadChip. In total, 24,273 CpG sites in 13,728 genes were analyzed, along with 33 human cell lines (Table S1). The iPSC line “201B7” was generated from human skin fibroblasts [8]. Quantitative scores of DNA methylation levels were obtained as β-values determined from the Illumina analysis, ranging from “0”, for completely unmethylated, to “1”, for completely methylated. We also performed genome-wide gene expression analysis using the Agilent Whole Human Genome Microarray chips. As assessed by unsupervised hierarchical clustering analysis and scatter plot of DNA methylation and gene expression data, human iPSCs could be clearly discriminated from their parent cells and were similar to ESCs (Figure 1C and Figure S5). The distribution of DNA methylation levels shows that the degree of global methylation in pluripotent stem cells was higher compared to the parent cells (Figure 1D, 1E), suggesting that a global gain of DNA methylation occurs during reprogramming.

### Identification of stem cell-specific differentially methylated regions (DMRs)

**Figure 2:**
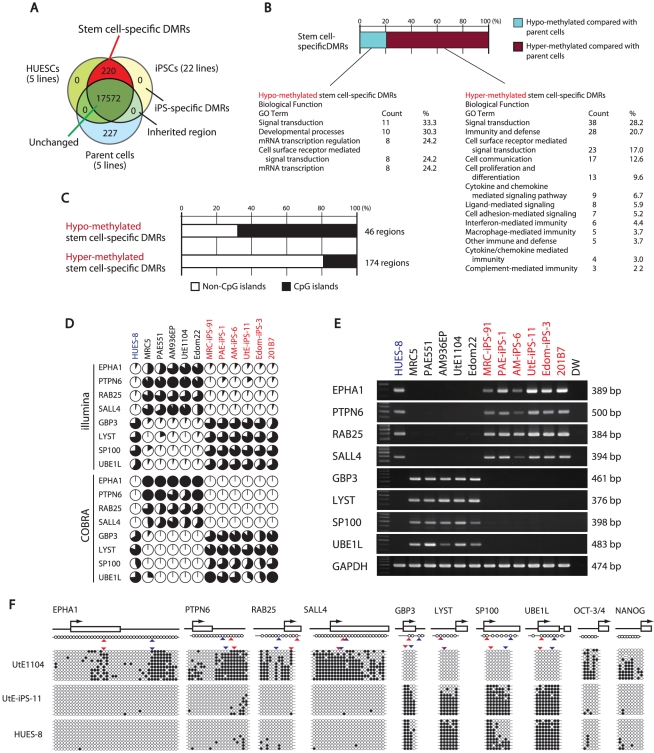
Defining stem cell-specific DMRs as novel epigenetic iPS markers. (*A*) Venn-like diagram showing overlapping CpG sites among ESCs, iPSCs and their parent cells. The 220 overlapping sites are stem cell-specific differentially methylated regions (DMRs). Notably, neither overlapping iPSCs-specific DMRs nor inherited regions in iPSCs from the parent cells were observed. (*B*) Proportion of the hyper- and hypo-methylated stem cell-specific DMRs and GO analysis. Approximately 80% of the regions were hyper-methylated in iPSCs, compared with that of the parent cells. (*C*) Proportion of the regions associated with CpG islands and non-CpG islands in the hypo-methylated stem cell-specific DMRs. The hypo-methylated regions were biased to CpG islands, whereas the hyper-methylated regions were biased to non-CpG islands. (*D*) DNA methylation levels in the 8 representative genes determined by Illumina Infinium HumanMethylation27 assay and Bio-COBRA. These 8 genes were defined as SS-DMRs with significant changes of expression and were described in Table S6. The relative amount of methylated and unmethylated DNA ratio is indicated as the black and white area, respectively, in the pie chart. (*E*) Expression of the 8 genes. Expression of the 8 genes had an inverse correlation with DNA methylation level. (*F*) Bisulfite sequencing analysis of the 8 genes in endometrial cells (UtE1104), UtE-iPS-11 and HUES-8 cells. (Top) Schematic diagram of the genes. Arrows, open boxes and open circles represent transcription start site, first exon and position of CpG sites, respectively. (Bottom) Open and closed circles indicate unmethylated and methylated sites, respectively. Red and blue arrowheads represent the position of CpG sites in Infinium assay and COBRA assay, respectively.

For further analysis, we defined DMR as representing a CpG site whose score differed 0.3 points or more from the β-value between the two groups. By comparison among ESCs (average from 5 lines), iPSCs (average from 22 lines), and parent cells (average from 5 lines), about 90% of the CpG sites (17,572 sites) examined did not show differential methylation among ESCs, iPSCs and parent cells (Figure 2A), suggesting that only a small number of the CpG sites is affected during reprogramming. The number of the CpG sites has been reported to be larger by genome-wide analysis [21].

We then identified 220 sites that are pluripotent stem cell-specific DMRs (Figure 2A). The 174 sites (79.5%) of the stem cell-specific DMRs had significantly higher methylation levels in iPSCs/ESCs when compared to the parent cells (Figure 2B). Approximately 80% of the DMRs between the iPSCs and their parent cells changed to a “hyper-methylated” state from a “hypo-methylated” state in iPSCs. In contrast, 45 sites of the stem cell-specific DMRs are hypo-methylated in iPSCs/ESCs, compared with the parent cells. Gene ontology analysis indicates that the hypo-methylated stem cell-specific DMRs especially included genes related to mRNA transcription regulation (Figure 2B). Interestingly, the majority of the hypo-methylated stem cell-specific DMRs were located on CpG islands, whereas the majority of the hyper-methylated stem cell-specific DMRs were located on non-CpG islands (Figure 2C). No iPS-specific DMRs were detected. We extracted 3,123 sites that are differentially methylated in one or more parent-specific iPSCs, compared to their parent cells, because DMRs are dependent on parent cell types (Figure S6). These DMRs are here designated as stem cell-required DMRs. Distribution analysis of the stem cell-required DMRs revealed a dispersed pattern rather than specific localization on the genome (Figure S7A).

From the combined gene expression and DNA methylation data, we chose 27 genes in the stem cell-specific DMRs showing more than a 5-fold change in expression of human iPSCs/ESCs, as compared with those in the parent cells (Table S4). Nine genes with hypo-methylated stem cell-specific DMRs were found in the group “genes significantly expressed in iPSCs/ESCs,” and 17 genes with hypo-methylated stem cell-specific DMRs belonged to the category “low expression or silenced in iPSCs/ESCs”. In addition, the methylation state and gene expression in *EPHA1*, *PTPN6*, *RAB25*, *SALL4*, *GBP3*, *LYST*, *SP100* and *UBE1L* were confirmed by quantitative combined bisulfite restriction analysis (COBRA) [25] (Figure 2D), RT-PCR (Figure 2E) and bisulfite sequencing (Figure 2F).

We also extracted genes with stem cell-required DMRs exhibiting high expression or suppression in human iPSCs/ESCs (Tables S5, S6). Interestingly, gene ontology analysis of the genes with stem cell-required DMRs showed that genes in the transcription factor category were detected only in the hypo-methylated stem cell-required DMRs (Table S7). The top 20 transcription factor genes with hypo-methylated stem cell-required DMRs exhibiting high expression in human iPSCs are summarized in Table 1 and include *OCT-4/3* (also known as *POU5F1*), *SALL4*, *SOX8*, *ZIC5*, and *FOXD1*.

**Table 1:** List of the top 20 out of 82 transcription factor genes with hypo-methylated stem cell-required DMRs exhibiting “high” expression in human iPS cells.

		DNA methylation		
TargetID	Gene name	HUESCs	iPSCs	Expression level
cg13083810	POU5F1, POU domain; class 5; transcription factor 1 isoform 1	0.584	0.549	55543.9
cg06303238	SALL4, sal-like 4	0.032	0.026	29766.2
cg16990174	RYBP, RING1 and YY1 binding protein	0.076	0.119	10274.1
cg03589001	MORF4L1, MORF-related gene 15 isoform 2	0.176	0.173	7015.7
cg02204046	MYCN, v-myc myelocytomatosis viral related oncogene; neuroblastoma derived	0.022	0.027	5826.8
cg10705800	CITED4, Cbp/p300-interacting transactivator; with Glu/Asp-rich carboxy-terminal domain; 4	0.438	0.445	5342.2
cg21696393	SOX8, SRY (sex determining region Y)-box 8	0.074	0.061	1976.7
cg23131007	TCF12, transcription factor 12 isoform b	0.138	0.155	1930.7
cg18808261	SATB1, special AT-rich sequence binding protein 1	0.194	0.242	1634.4
cg15607672	OTX2, orthodenticle 2 isoform a	0.046	0.054	1227.5
cg05345286	MDFI, MyoD family inhibitor	0.023	0.040	1035.9
cg20909686	OVOL1, OVO-like 1 binding protein	0.215	0.204	991.0
cg26209676	ZNF581, zinc finger protein 581	0.113	0.196	916.1
cg05522383	PITX2, paired-like homeodomain transcription factor 2 isoform b	0.024	0.030	544.8
cg17675150	ZNF532, zinc finger protein 532	0.069	0.107	525.3
cg01510051	ZNF542, zinc finger protein 542	0.585	0.555	443.9
cg06154570	HEYL, hairy/enhancer-of-split related with YRPW motif-like	0.134	0.152	440.3
cg12556134	TGIF2, TGFB-induced factor 2	0.075	0.072	405.4
cg03663715	FOXD1, forkhead box D1	0.030	0.042	349.1
cg09721427	HHEX, hematopoietically expressed homeobox	0.077	0.101	206.9

“Expression level” is an average of raw data values in iPSCs from Gene Chip data.

### Aberrant and inherited methylation in iPSCs

**Figure 3:**
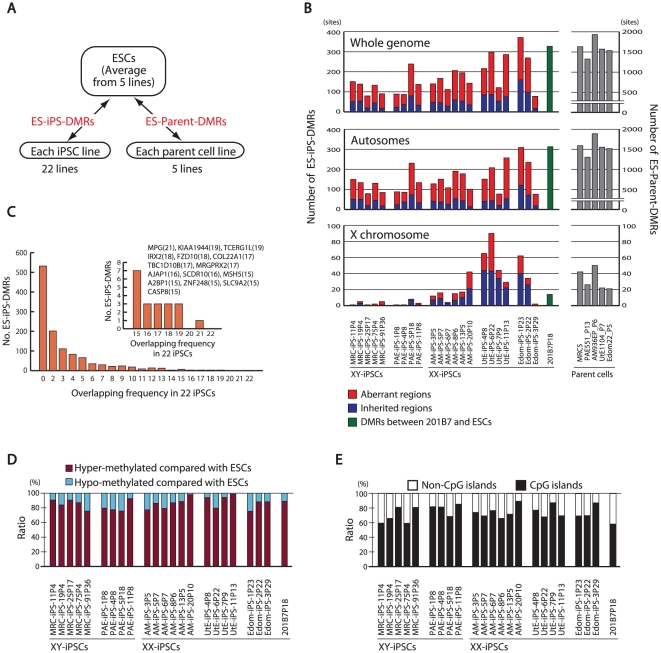
Aberrant methylation in human iPSCs. (*A*) Comparison of DNA methylation states of each iPSC line or each parent cell line with that of ESCs. The DMRs between ESCs and iPSCs are designated as ES-iPS-DMRs, and the DMRs between ESCs and parent cells are designated as ES-parent-DMRs. (*B*) The number of ES-iPS-DMRs and ES-parent-DMRs on whole genome (top), autosomes (middle) and X chromosome (bottom). Ratios of number of inherited regions in iPSCs from parent cells (blue) and aberrant regions in iPSCs that differ from ESCs and parent cells (red) in the ES-iPS-DMRs are shown in bars. Female iPSCs were demonstrated to carry high number of EiP-DMRs on X chromosome. (*C*) Number of overlapped ES-iPS-DMRs frequency in iPSCs. No overlapping ES-iPS-DMRs in all 22 iPSC lines. (Inlet) A small number of overlapping ES-iPS-DMRs of the frequency from 15 to 22. Overlapping frequency of each gene is indicated in parentheses. (*D*) Proportion of the hyper- and hypo-methylated ES-iPS-DMRs. More than 75% of the ES-iPS-DMRs were hyper-methylated in iPSCs. (*E*) Proportion of the ES-iPS-DMRs associated with CpG islands and non-CpG islands in ach iPSC line. ES-iPS-DMRs were biased to CpG islands.

Few changes in DNA methylation were detected between iPS and ES cells and these were not consistent among the different iPS lines (Figure 2A, Figures S6, S7). In further analyses, we compared the DNA methylation states of each iPSC line or each parent cell line with that of ESCs (averaged value) (Figure 3A). For the whole genome, the number of DMRs between ESCs and iPSCs (ES-iPS-DMRs) varied in the 22 iPSC lines (Figure 3B). A comprehensive analysis of methylation in ESCs and iPSCs identified 1,459 ES-iPS-DMRs covering 1,260 genes that were differentially methylated in one or more iPSC lines. ES-iPS-DMRs are composed of aberrant (iPS-specific) methylation sites, in comparison with ESCs and inherited methylation sites from the parent cells. The number of inherited sites as well as aberrant sites varied among iPSCs. Analysis of the ES-iPS-DMRs on each chromosome showed a characteristic distribution of the ES-iPS-DMRs on the X chromosome in XX-iPSCs (Figure 3B and Figure S8). Female XX-iPSCs demonstrate a tendency to carry a large number of ES-iPS-DMRs on the X chromosome, but male XY-iPSCs had few ES-iPS-DMRs on the X chromosome (Figure 3B, lower panel). While no ES-iPS-DMRs overlapped for all the iPSCs (Figure 2A), 20 ES-iPS-DMRs overlapped in more than 15 out of 22 lines (Figure 3C, inset). These 20 ES-iPS-DMRs include the genes for *MPG* (N-methylpurine-DNA glycosylase isoform b), *FZD10* (frizzled 10), *IREX2* (iroquois homeobox protein 2) and *ZNF248* (zinc finger protein 248), which are highly associated with aberrant methylation during reprogramming. Distribution analysis of the ES-iPS-DMRs across the genome did not show any specific localization (Figure S9). We further compared overlapping ES-iPS-DMRs in reference to a genome-wide methylation analysis [21], and found that 72 gene promoters overlapped between our data and that of Lister et al..

More than 70% of the ES-iPS-DMRs were hyper-methylated in each iPSC (Figure 3D), indicating that the iPSC genome is more methylated than the ESC genome. In addition, the majority of the ES-iPS-DMRs were located on CpG islands (Figure 3E), suggesting that aberrant methylation is biased towards CpG islands.

### Effect of long-term culture on DNA methylation status in iPSCs

**Figure 4:**
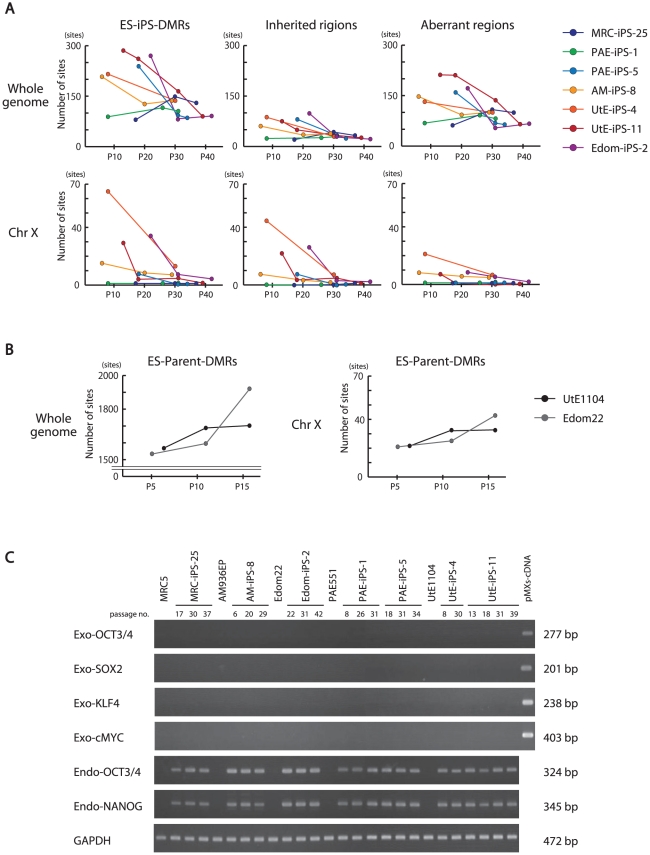
Effect of long-term cultivation on ES-iPS-DMRs. (*A*) Decrease in the number of the ES-iPS-DMRs with continuous passaging. Upper panels show change of the number of the ES-iPS-DMRs (left), the inherited regions (middle) and aberrant regions (right) on whole genome. Lower panels show change in the number of the ES-iPS-DMRs (left), inherited regions (middle) and aberrant regions (right) on X chromosome. The number of the ES-iPS-DMRs in XX-iPSCs approached zero with continuous passaging on X chromosome. In contrast, XY-iPSCs had few ES-iPS-DMRs on X chromosome throughout the passages. (*B*) The number of the ES-parent-DMRs with continuous passaging. (*C*) No expression of the transgenes in iPSCs at each passage was detected by RT-PCR.

We investigated the effect of continuous passaging on the DNA methylation profile of human iPSCs. To address the effect, we subjected 7 iPSC lines to additional rounds of passaging under identical culture conditions, and obtained genomic DNA and RNA at passage 4 (P4) to P40 for DNA methylation and gene expression. The number of the ES-iPS-DMRs ranged from 80 in MRC-iPS-25 to 286 in UtE-iPS-11 at early passage (P10 to P20), whereas the number of the ES-iPS-DMRs dramatically decreased in all lines at late passage (P30 to P40) (Figure 4A, upper-left panel). The number of inherited and aberrant sites decreased to 30 and 70, respectively, at P30 to P40 (Figure 4A, upper-center and right panels). These decreases in the numbers of ES-iPS-DMRs indicate that iPSCs have become closer to ESCs in their DNA methylation profiles. In particular, XX-iPSC lines (AM-iPS-8, UtE-iPS-4 and -11, and Edom-iPS-2) showed decreases in the number of ES-iPS-DMRs with passaging. The XY-iPSC lines, such as MRC-iPS-25 and PAE-iPS-1, had only a small number of ES-iPS-DMRs. The number of ES-iPS-DMRs continued to decrease to approximately 100 ES-iPS-DMRs containing 30 inherited sites. Intriguingly, few ES-iPS-DMRs on the X chromosome were detected in XY-iPSCs throughout the passaging. In contrast, the number of ES-iPS-DMRs in XX-iPSCs ranged from 10 to 70 at the early passage (P4 to P20), and decreased to zero after P30 (Figure 4A, lower panels). We also investigated the effect of continuous passaging on the DNA methylation profile of the parent cells (UtE1104 and Edom22) (Figure 4B). The number of the DMRs between ESCs and parent cells (ES-parent-DMRs) increased with passaging. In addition, we also confirmed that the transgenes were silenced at each passage (Figure 4C and Figure S4), indicating that the decreasing number of the ES-iPS-DMRs in iPSCs occurred in the transgene-independent phase.

### Comparative analysis of ES-iPS-DMRs dynamics

**Figure 5:**
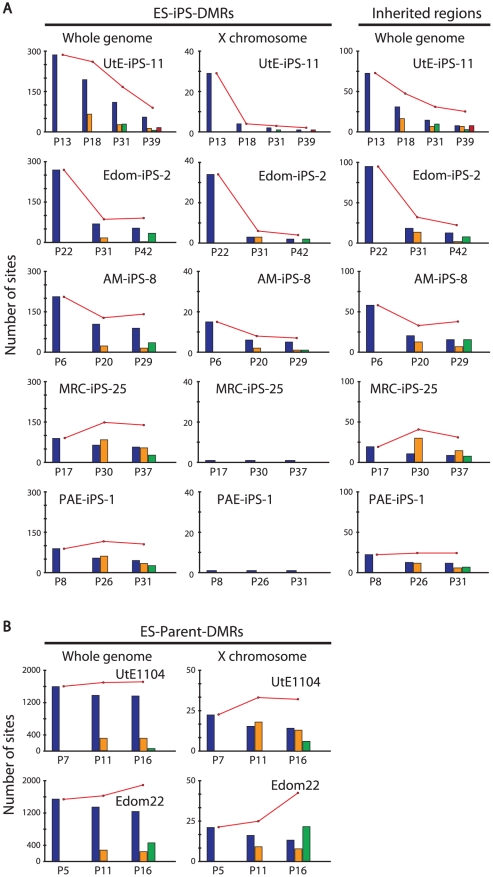
Number of the ES-iPS-DMRs and ES-parent-DMRs with passaging. (*A*) Number of the ES-iPS-DMRs with passaging. Red line plots indicate total number of the ES-iPS-DMRs. Blue bars indicate the number of the ES-iPS-DMRs that appeared at the earliest passage. Orange, green and red bars indicate the number of the ES-iPS-DMRs that appeared secondarily at later passages. Appearance/disappearance of the ES-iPS-DMRs and inherited regions were repeated, but the number of newly-appeared ES-iPS-DMRs was decreased with passaging. (*B*) Number of the ES-parent-DMRs with passaging. Blue bars indicate the number of the ES-parent-DMRs at P5 (or P7). Orange and green bars indicate de novo ES-parent-DMRs at P11 and P16, respectively.

We then compared each ES-iPS-DMRs with passaging. The UtE-iPS-11 had 286 ES-iPS-DMRs at P13, 194 sites at P18, 110 sites at P31, and 55 sites at P39. The ES-iPS-DMRs detected at P13 decreased with passaging (blue bars in upper-left panel in Figure 5A). Interestingly, 66 de novo ES-iPS-DMRs appeared at P18, while at P13 these sites showed no differences between UtE-iPS-11 and ESCs (orange bars in upper-left panel in Figure 5A). These 66 ES-iPS-DMRs also decreased with passaging (P31 and P39). The 29 additional ES-iPS-DMRs at P31 also appeared and decreased with passaging (P39) (green bars in upper-left panel in Figure 5A) and 16 ES-iPS-DMRs at P39 (red bar in upper-left panel in Figure 5A) appeared. Rapid appearance and gradual disappearance of ES-iPS-DMRs was a recurring theme, but the number of newly-appearing ES-iPS-DMRs decreased with passaging (Figure 5A, upper-left panel). The same change in ES-iPS-DMRs occurred on the X chromosome, but the number of the ES-iPS-DMRs approached zero at early passages (Figure 5A, upper-center panel). Intriguingly, this change also occurred at inherited sites, which was contrary to our expectations. The inherited sites also repeatedly appeared and disappeared, and the number of newly-appearing inherited sites decreased with passaging (Figure 5A, upper-right panel). The term “inherited” is here used to mean the same methylation state found in iPSCs and their parent cells, but the “inherited” regions behaved like “aberrant” regions that had multiple appearances and disappearances. These multiple appearances/disappearances of ES-iPS-DMRs were observed in all iPSC lines regardless of parental cell type. The ES-parent-DMRs were also analyzed. The *de novo* ES-parent-DMRs appeared as well as the ES-iPS-DMRs, but did not decrease with passaging (Figure 5B).

### Most ES-iPS-DMRs were hyper-methylated in iPSCs

**Figure 6:**
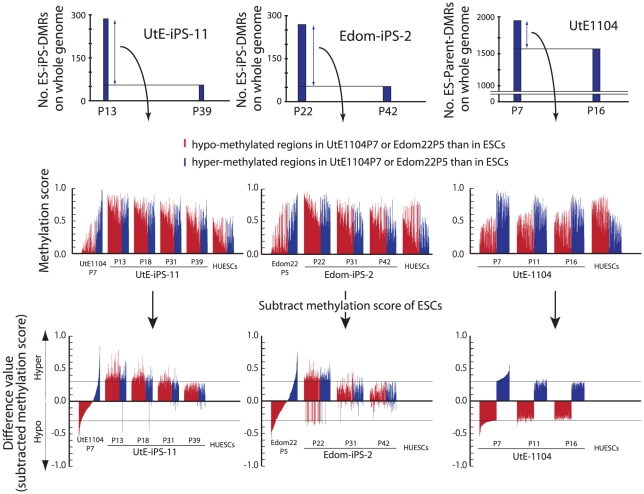
Hyper-methylation in the ES-iPS-DMRs and ES-parent-DMRs. ES-iPS-DMRs that disappeared in UtE-iPS-11 and Edom-iPS-2 at the latest passage (upper) were analyzed and the methylation score of each ES-iPS-DMR was plotted on bar graph (middle). To clearly compare methylation scores, difference value were estimated by subtracting the scores of ESCs from that of each sample (lower). Red and blue bars represent hypo- and hyper-methylated regions, respectively, in the parent cells, compared with ESCs. Notably, almost all the regions, even though their difference values were hypo-methylated in the parent cells, became hyper-methylated in iPSCs at the early passage, and then their methylation levels were adjusted to the level of ESCs with passaging, i.e. subtracted methylation score became close to zero. This transiently-induced hyper-methylation was not detected in parent cells.

ES-iPS-DMRs can be categorized into two groups: a, hyper-methylated and b, hypo-methylated sites in iPSCs, as compared with ESCs. ES-iPS-DMRs that disappeared at the last passage (P39) (blue bars in Figure 5) in both UtE-iPS-11 and Edom-iPS-2 were extracted, and each methylation score of the extracted ES-iPS-DMRs is shown (Figure 6, upper and middle panels). To compare methylation scores, a “difference value” was estimated by subtracting the scores of ESCs from those of each cell (Figure 6, lower panels). Positive and negative difference values indicate that these sites are hyper- and hypo-methylated, respectively, when compared with ESCs. Difference values of the ES-iPS-DMRs showing aberrant methylation states in iPSCs at the early passage approached zero with passaging. It should be noted that the almost all difference values became largely positive in iPSCs at early passage (P13 or P22), even though they were negative in the parent cells, and then approached zero upon further passaging. This transiently-induced hyper-methylation was observed at each passage in all iPSC lines examined. The observed transient hypermethylation patterns during iPS reprogramming did not correspond to methyflated CpGs in the parental cells. However, this observation does not rule out that transient aberrant methylation could also be observed in some cases on sites that were methylated in the parental cells.

## Discussion

### Identification of novel epigenetic iPS markers

*OCT-4/3* and *NANOG* have been used as epigenetic markers for iPSCs [8]–[10], [26], [27]. We previously showed candidate epigenetic markers by analyzing 6 iPS lines [17]. Here we identified 8 novel epigenetic markers more closely by defining 9 genes with the hypo-methylated stem cell-specific DMRs and significantly higher expression, and 17 genes with the hyper-methylated stem cell-specific DMRs and significantly lower expression in iPSCs/ESCs from 22 iPS lines. DNA methylation and expression of these genes, especially the 8 genes, *SALL4*, *EPHA1*, *PTPN6*, *RAB25*, *GBP4*, *LYST*, *SP100* and *UBE1L*, can now be used as epigenetic markers for pluripotent stem cells. Among these 8 genes, *SALL4* has been used as an expression marker, and is revealed for the first time as an epigenetic marker. These epigenetic changes during reprogramming can be detected by 3 different methods (Illumina assay, COBRA and bisulfite sequencing), and is evident, i.e. CpG sites are methylated or unmethylated in an all-or-none fashion. The identification of these novel epigenetic markers can be another tool for the validation of pluripotent stem cells that are iPSCs and ESCs.

The hypo-methylated stem cell-required DMRs may have an important role for reprogramming as do the stem cell-specific DMRs, because reprogramming is dependent on the type of parent cells. In fact, genes associated with the hypo-methylated stem cell-required DMRs include a large number of transcription factors that are involved in pluripotency. Establishment of the stem cell-required DMRs database in iPSCs derived from different types of parent cells can help to generate human iPSCs in a fast and easy manner. Hypo-methylated stem cell-specific regions have been reported to be abundant in CpG islands [28]–[30]. In this study, the hypo-methylated stem cell-specific DMRs were significantly biased towards CpG islands, whereas the hyper-methylated stem cell-specific DMRs were biased to non-CpG islands, suggesting that genes with CpG islands have a propensity to be demethylated during reprogramming towards pluripotent stem cells. The higher number of the hyper-methylated stem cell-specific DMRs in iPSCs indicates that the Yamanaka factors activate only limited numbers of stem cell-specific/associated genes through demethylation of the specific DMRs shown in this study on the genome in parallel with methylating most genes associated with tissue-specific function during reprogramming.

### Multiple appearances/disappearances of aberrant hyper-methylation

**Figure 7:**
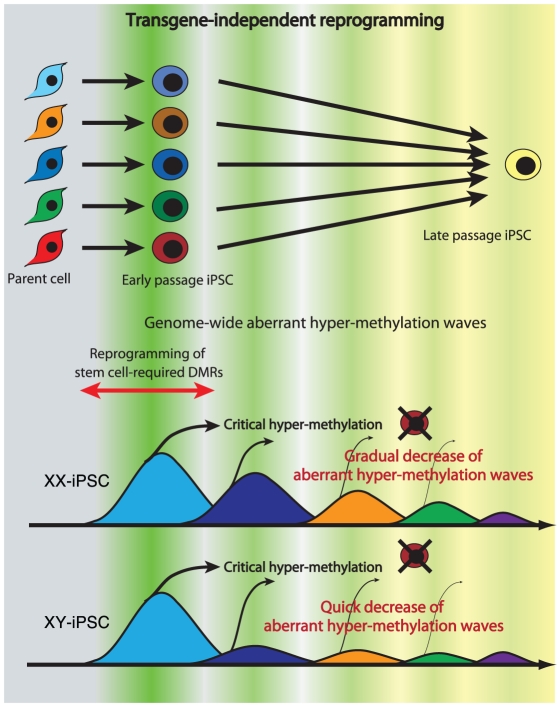
Model of mechanism for transgene-independent reprogramming. During reprogramming from somatic cells to iPSCs, the cells undergo dynamic change of methylation of SS-DMRs and genome. The cells with incomplete reprogramming or excessive hyper-methylation of the genome fail to maintain pluripotency at early passages. Human iPSCs are transgene-independently reprogrammed gradually through “convergence” of periodic aberrant hyper-methylation and become closer to ESCs upon continuous passaging. Due to the sensitivity to aberrant methylation on X chromosome, XY-iPSCs become close to ESCs faster than XX-iPSCs do.

Continuous passaging of iPSCs reduces differences among clones in gene expression profiles in mouse [15] and in human [31] cells. Here we detected multiple appearances and disappearances of aberrant hyper-methylation throughout iPSC reprogramming. Furthermore, human iPSCs were gradually reprogrammed through the “convergence” of periodic aberrant hyper-methylation upon continuous passaging (Figure 7). The term “convergence” is used here to mean that amplitude of aberrant hyper-methylation (or number of ES-iPS-DMRs) decreases. The decrease of aberrant methylation suggests that iPSCs lose the characteristics inherited from the parent cells and adapt to ESCs. This aberrant and stochastic hyper-methylation and their convergence may be a direct cause of the transgene-independent phases of iPS reprogramming [15]. Aberrant hyper-methylation, for which the mechanism remains unclear, can possibly be attributed, at least in part, to up-regulation of DNMT3B, a de novo methyltransferase, at the early stages of reprogramming.

Maintenance of an epigenetic memory of their parent cells at early passage of human iPSCs (Figure 4A) is consistent with recent reports involving mouse iPSCs [15]–[17]. However, most inherited sites from the parent cells in iPSCs were inconsistent among iPSC clones from the same parent cells on the genome, and these sites showed periodic aberrant hyper-methylation during passaging, as well as aberrant sites. Inherited methylation is non-synchronous and stochastic, much like aberrant methylation, rather than deterministic. The inherited sites thus comprise a portion of all aberrant methylation observed in the clones.

Mouse female iPSCs as well as mouse female ESCs carry two active X chromosomes [32], but inactivation of the X chromosome in human female ESCs is variable [22], [33]–[35]. It has been reported recently that human female iPSCs show a variable state of X-inactivation as is seen in human female ESCs [22], [36]. In this study, human iPSCs exhibited a dynamic epigenetic state on the X chromosome. The ES-iPS-DMRs on the X chromosome in XY-iPSCs were rare and the average number of ES-iPS-DMRs in XY-iPSCs was significantly lower than in XX-iPSCs, suggesting that iPSCs are prone to aberrant hyper-methylation on the inactive X chromosome. A recent report showed that X inactivation in human ESCs is sensitive to the level of oxygen through culture in vitro [35]. Therefore, analysis of aberrant methylation in iPSCs that are established and cultured in low oxygen condition would be help to understand physiological relevance of X inactivation and reprogramming.

### Incomplete adaptation of iPSCs to ESCs

The number of passages for “convergence” of the aberrant hyper-methylation seems be dependent on parental cell types and their sex. Disappearance of iPSCs in culture within 10 passages is occasionally observed, regardless of the cell of origin. This instability may be due to an excess of aberrant hyper-methylation at early passages in addition to the “partial reprogramming” theory [15]. The late-passage iPSCs, like the early-passage iPSCs, retained the ability to differentiate into cell types found in all three germ layers. iPSCs showed reduced aberrant methylation during adaptation to ESCs; however, iPSCs retained approximately 100 aberrant sites on autosomes, implying that iPSCs do not become identical to ESCs, although they become very close. The remaining aberrant sites were inconsistent among iPSC clones with different parent cell types, but the numbers were consistent among iPSC clones after a 42-week cultivation. The quantity (or number) of ES-iPS-DMRs would be another validation index for iPSC identity as well as quality analysis (or methylation ratio) of pluripotent stem cell-specific methylation.

### Abnormalities of imprint genes, *MEG3* genes, and *H19* genes in human iPSCs

Genomic imprinting of *H19*, *IGF2* and *MEG3* has been reported to be unstable in human ESCs [37], [38]. The *Dlk1*-*Dio3* genes were aberrantly silenced in most of the mouse iPSC lines. But mouse iPSCs without *MEG3* expression still have the ability to differentiate into cell type of three germ layers *in vitro* [39]. In humans, IG-DMR and MEG3-DMR are relevant to upd(14)pat-like and upd(14)mat-like phenotypes [40]. In this study, only *MEG3* and *H19*, out of 87 imprinted genes examined showed aberrant methylation in human iPSCs (Figure S10). Six out of 15 human iPSC lines were aberrantly methylated at MEG3-DMR. *MEG3* expression was silenced in those six lines regardless of their parent cell type, although all parent cells showed about 50% methylation at MEG3-DMR and expression of *MEG3* (Figure S10A, S10B). However, MEG3-negative iPS lines are almost indistinguishable from MEG3-positive iPS lines in DNA methylation and gene expression in human. Continuous passaging did not resolve the aberrant hyper-methylation at MEG3-DMR, suggesting that these abnormalities occur at early passage and are fixed at later stages. In addition, aberrant hyper-methylation at *H19* in all iPSCs and ESCs was observed (Figure S10C), and *H19* was not expressed in all iPSCs and their parent cells.

We revealed that transgene-independent reprogramming is a convergence of periodic hyper-methylation. The aberrant hyper-methylation in iPSCs occurs stochastically throughout the genome. Early-stage iPSC clones with different propensities due to stochastic hyper-methylation may be used after selection of desirable phenotypes to treat a wide range of target diseases using cell-based therapy, and would thus have advantages for clinical use. In this sense, the number of ES-iPS-DMRs and methylation states of the stem cell-specific DMRs are useful epigenetic indices for evaluating human iPSCs in therapeutic applications.

## Materials and Methods

### Ethics statement

Human endometrium, amnion, placental artery endothelium and menstrual blood cells were collected by scraping tissues from surgical specimens, under signed informed consent, with ethical approval of the Institutional Review Board of the National Institute for Child Health and Development, Japan. Signed informed consent was obtained from donors, and the surgical specimens were irreversibly de-identified. All experiments handling human cells and tissues were performed in line with Tenets of the Declaration of Helsinki.

### Human cell culture

Endometrium (UtE1104), amnion (AM936EP), placental artery endothelium (PAE551) and menstrual blood cell (Edom22) cell lines were independently established in our laboratory [41], [42]. UtE1104, AM936EP, Edom22, and MRC-5 [43] cells were maintained in the POWEREDBY10 medium (MED SHIROTORI CO., Ltd, Tokyo, Japan). PAE551 cells were cultured in EGM-2MV BulletKit (Lonza, Walkersville, MD, USA) containing 5% FBS. Human iPSCs were generated in our laboratory, via procedures described by Yamanaka and colleagues [8] with slight modification [17], [41], [44]–[46]. The human cells were infected with retroviruses produced from the retroviral vector pMXs, which encodes the cDNA for human *OCT3/4*, *SOX2*, *c-MYC*, and *KLF4*. Human iPSCs were established from MRC-5, AM936EP, UtE1104, and PAE551, which were designated as MRC-iPSCs, AM-iPSCs, UtE-iPSCs and PAE-iPSCs [17], [41], [44]–[46]. Edom- iPSCs were established from Edom22 in this study. Human iPSCs were maintained on irradiated MEFs in 0222 medium (MED SHIROTORI CO., Ltd, Tokyo, Japan) supplemented with 10 ng/ml recombinant human basic fibroblast growth factor (bFGF, Wako Pure Chemical Industries, Ltd., Osaka, Japan). The 201B7 human iPSC line [8] that was generated from human skin fibroblasts by retroviral transfection with 4 transcription factors was also used. Frozen pellets of human ESCs (HUESCs) [23], [24] were kindly gifted from Drs. C. Cowan and T. Tenzan (Harvard Stem Cell Institute, Harvard University, Cambridge, MA).

### DNA methylation analysis

DNA methylation analysis was performed using the Illumina infinium assay with the HumanMethylation27 BeadChip (Illumina) and the BeadChip was scanned on a BeadArray Reader (Illumina), according to the manufacturer's instructions. Methylated and unmethylated signals were used to compute a β-value, which was a quantitative score of DNA methylation levels, ranging from “0”, for completely unmethylated, to “1”, for completely methylated. On the HumanMethylation27 BeadChip, oligonucleotides for 27,578 CpG sites covering more than 14,000 genes are mounted, mostly selected from promoter regions. CpG sites with ≥0.05 “Detection p value” (computed from the background based on negative controls) were eliminated from the data for further analysis, leaving 24,273 CpGs (13,728 genes) valid for use with the 51 samples tested. Average of methylation was calculated from HUESCs, MRC-iPSCs, AM-iPSCs, UtE-iPSCs, PAE-iPSCs and Edom-iPSCs, in which DMRs among each line in the each set were removed. Analyzed data sets (list of stem cell-specific DMRs and stem cell-required DMRs) can be obtained from http://www.nch.go.jp/reproduction/e/thdmds.html.

### Gene expression analysis

Gene expression analysis was performed using the Agilent Whole Human Genome Microarray chips G4112F (Agilent, Santa Clara, CA), which contains over 41,000 probes. Raw data were normalized and analyzed by GeneSpringGX11 software (Silicon Genetics, Redwood City, CA). For RT-PCR, an aliquot of total RNA was reverse-transcribed using Random Hexamer primers. The cDNA template was amplified using specific primers for *EPHA1*, *PTPN6*, *RAB25*, *SALL4*, *GBP3*, *LYST*, *SP100*, *UBE1L*, OCT3/4 and NANOG. For detecting RNA derived from transgenes, specific primer sets, FY-11 and OCT3/4-SR, FY-11 and SOX2-SR, KLF4-SF and FY-12, cMYC-SF and FY-12, were used. Expression of glyceraldehyde-3-phosphate dehydrogenase (GAPDH) was used as a control. Primers used in this study are summarized in Table S8.

### Quantitative combined bisulfite restriction analysis (COBRA) and bisulfite sequencing

To confirm the DNA methylation state, bisulfite PCR-mediated restriction mapping (known as the COBRA method) was performed. Sodium bisulfite treatment of genomic DNA was carried out using EZ DNA Methylation-Gold kit (Zymo Research). PCR amplification was performed using BIOTAQ HS DNA polymerase (Bioline Ltd; London, UK) with specific primers for *EPHA1*, *PTPN6*, *RAB25*, *SALL4*, *GBP3*, *LYST*, *SP100*, and *UBE1L*. Primers used in this study are summarized in Table S8. After digestion with restriction enzymes, HpyCH4IV or Taq I, quantitative-COBRA coupled with the Shimadzu MCE-202 MultiNA Microchip Electrophoresis System (Shimadzu, Japan) was carried out for quantitative DNA methylation level. To determine the methylation state of individual CpG sites, the PCR product was gel extracted and subcloned into pGEM T Easy vector (Promega, Madison, WI), and then sequenced. The promoter regions of the *OCT3/4* and *NANOG* [41], [44] were also amplified and sequenced. Methylation sites were visualized and quality control was carried out by the web-based tool, “QUMA” (http://quma.cdb.riken.jp/) [47].

### Web tools

The following web tools were used in this study: NIA Array [48] (http://lgsun.grc.nia.nih.gov/ANOVA/) for hierarchical clustering, DAVID Bioinformatics Resources [49] (http://david.abcc.ncifcrf.gov/home.jsp), PANTHER Classification System [50] (http://www.pantherdb.org/).

### Accession numbers

NCBI GEO: HumanMethylation27 BeadChip data and gene expression microarray data have been submitted under accession number GSE 20750, GSE24676 and GSE24677.

## Supporting Information

Figure S1 Immunohistochemistry of stem cell-specific surface antigens, NANOG, OCT3/4, SOX2, SSEA-4 and TRA-1-60 in AM-iPSCs, MRC-iPSCs and Edom-iPSCs, and teratoma formation of those iPSCs by subcutaneous implantation into NOD/Scid mice. The iPSCs differentiated to various tissues including ectoderm (neural tissues and retinal pigment epithelium), mesoderm (cartilage) and endoderm (gut). Immunostaining and teratoma formation were carried out as previously described [41], [44].
(PDF)

Figure S2 Immunohistochemistry of stem cell-specific surface antigens, NANOG, OCT3/4, SOX2, SSEA-4 and TRA-1-60 in PAE-iPSCs and UtE-iPSCs, and teratoma formation of those iPSCs by subcutaneous implantation into NOD/Scid mice. The iPSCs differentiated to various tissues including ectoderm (neural tissues and retinal pigment epithelium), mesoderm (cartilage) and endoderm (gut). Immunostaining and teratoma formation were carried out as previously described [41], [44].
(PDF)

Figure S3 Bisulfite sequencing at the OCT3/4 and NANOG promoter regions in ESCs, iPSCs and their parent cells.
(PDF)

Figure S4 Expression of the transgenes in iPSCs. (A) RT-PCR for transgenes in 22 iPSC lines. No expression of the transgenes in each iPSC lines was detected. (B) Quantitative RT-PCR for the transgenes at each passage. Relative expression of each transgene normalized to GAPDH was calculated. P0(D2), RNA from UtE1104 cells that were infected with the retroviruses and were cultured for 2 days. No expression of the transgenes at each passage was detected.
(PDF)

Figure S5 (A) Unsupervised hierarchical clustering analysis based on DNA methylation (left) and gene expression (right) in each ESC line, iPSC line and their parent cell line. (B) Unsupervised hierarchical clustering analysis based on DNA methylation (left) and gene expression (right) of average of ESCs, iPSCs and parent cells. (C) Scatter plot of DNA methylation (left) and gene expression data (right) in ESCs, iPSCs and their parent cells.
(PDF)

Figure S6 (A) Venn-like diagram showing seven categories (aa-gg) overlapped CpG sites among ESCs, iPSCs and their parent cells. (B) Number of CpG sites involved in each seven category from the five ESCs-iPSCs-the parent cell sets. “Overlapped” indicates a number of sites that overlap in all iPSCs examined. The 220 overlapping sites in “ee” are designated as stem cell-specific differentially methylated regions (DMRs) and 3,123 total sites in “ee” are designated as stem cell-required DMRs. Notably, no overlapping sites were observed in “bb” that is a category involved in iPSCs-specific DMRs and in “ff” that is a category involved in inherited regions in iPSCs from the parent cells.
(PDF)

Figure S7 (A) Distribution of stem cell-required DMRs on each chromosome (upper) and frequency on each chromosome (bottom). (B) The number of parent cell specific DMRs (left) and the number of iPSC derived from different parent cells specific DMRs (left).
(PDF)

Figure S8 The number of DMRs between ESCs and each iPSC line (ES-iPS-DMRs) on each chromosome. ES-iPS-DMRs between 201B7 (iPSCs from Yamanaka) and ESCs are shown for comparison.
(PDF)

Figure S9 Distribution of the ES-iPS-DMRs on each chromosome. Distribution of the EiP-DMRs overlapped in less than 9 lines (light blue bars), in more than 10 and less than 14 lines (blue bars), and in more than 15 lines (red bars) among 22 lines.
(PDF)

Figure S10 DNA methylation at human *MEG3* and *H19*. (A) DNA methylation at MEG3-DMR (CG7) and expression of MEG3. (Top) Schematic diagram of the MEG3 gene. The arrow, open boxes and open circles represent transcription start site, first exon and position of CpG sites, respectively. Red and blue arrowheads represent the position of CpG sites in Infinium assay and COBRA assay, respectively. DNA methylation scores of MEG3 were determined by Illumina Infinium HumanMethylation27 assay (upper bar graph) and Bio-COBRA (lower bar graph). (Bottom) Expression of MEG3 and GAPDH was determined by RT-PCR. Information of MEG3 primers for COBRA and RT-PCR is described by Kagami et al. [40]. (B) Bisulfite sequencing analysis of MEG3-DMRs (CG7). (C) Methylation scores of H19 were determined by Illumina Infinium HumanMethylation27 assay.
(PDF)

Table S1 List of human cells analyzed for a methylation state in this study.
(PDF)

Table S2 STR analysis of iPSCs.
(PDF)

Table S3 Karyotypic analysis of iPSCs.
(PDF)

Table S4 List of genes with stem cell-specific DMRs exhibiting significant changes in expression in human iPS cells.
(PDF)

Table S5 List of the top 100 genes with hypo-methylated stem cell-required DMRs exhibiting ‘high’ expression in human iPS cells.
(PDF)

Table S6 List of top 100 genes with hyper-methylated stem cell-required DMRs exhibiting suppression in human iPS cells.
(PDF)

Table S7 List of top 5 categories of GO Term in “Stem cell-required DMRs”.
(PDF)

Table S8 Primer list.
(PDF)
