# Supplementary figures and images for: DNA Methylation Dynamics in Human Induced Pluripotent Stem Cells over Time
Source: PLoS Genet. 2011 May 26;7(5):e1002085. doi: 10.1371/journal.pgen.1002085 (PMC3102737; doi:10.1371/journal.pgen.1002085)

Figure S1

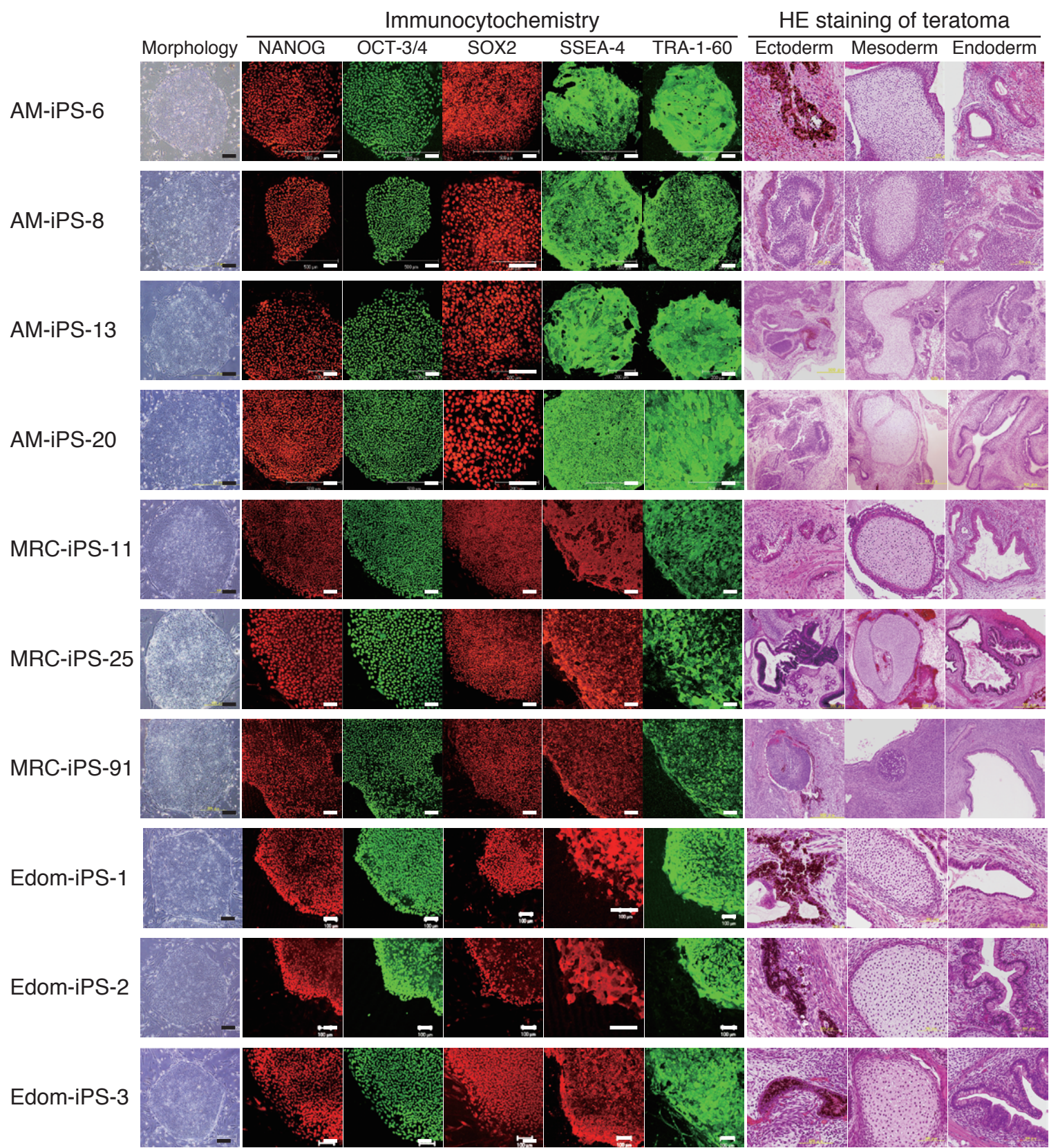

Supplement: Figure S1 — Immunohistochemistry of stem cell-specific surface antigens, NANOG, OCT3/4, SOX2, SSEA-4 and TRA-1-60 in AM-iPSCs, MRC-iPSCs and Edom-iPSCs, and teratoma formation of those iPSCs by subcutaneous implantation into NOD/Scid mice. The iPSCs differentiated to various tissues including ectoderm (neural tissues and retinal pigment epithelium), mesoderm (cartilage) and endoderm (gut). Immunostaining and teratoma formation were carried out as previously described [41], [44]. (PDF) [file pgen.1002085.s001.pdf]

Figure S2

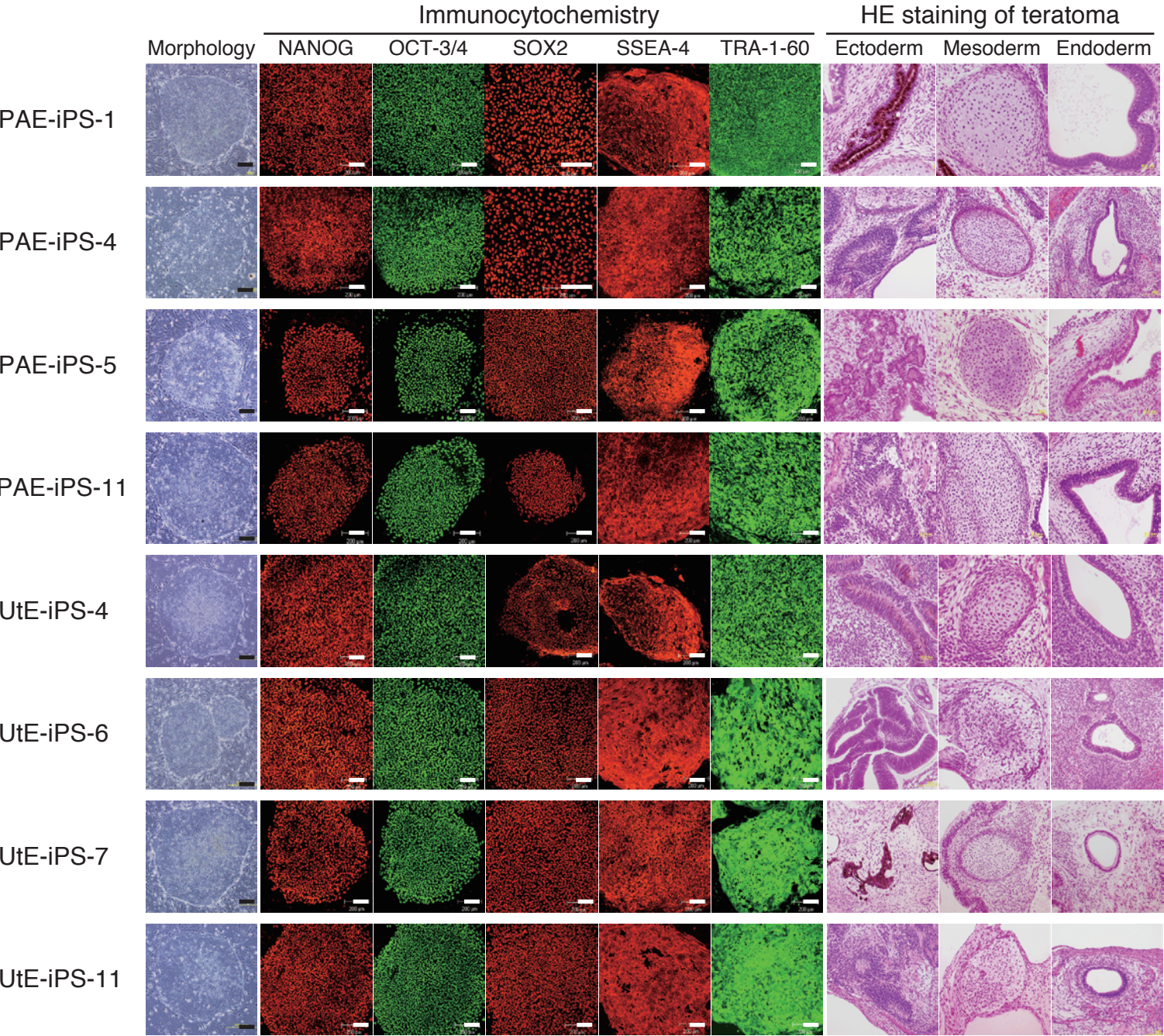

Supplement: Figure S2 — Immunohistochemistry of stem cell-specific surface antigens, NANOG, OCT3/4, SOX2, SSEA-4 and TRA-1-60 in PAE-iPSCs and UtE-iPSCs, and teratoma formation of those iPSCs by subcutaneous implantation into NOD/Scid mice. The iPSCs differentiated to various tissues including ectoderm (neural tissues and retinal pigment epithelium), mesoderm (cartilage) and endoderm (gut). Immunostaining and teratoma formation were carried out as previously described [41], [44]. (PDF) [file pgen.1002085.s002.pdf]

Figure S3

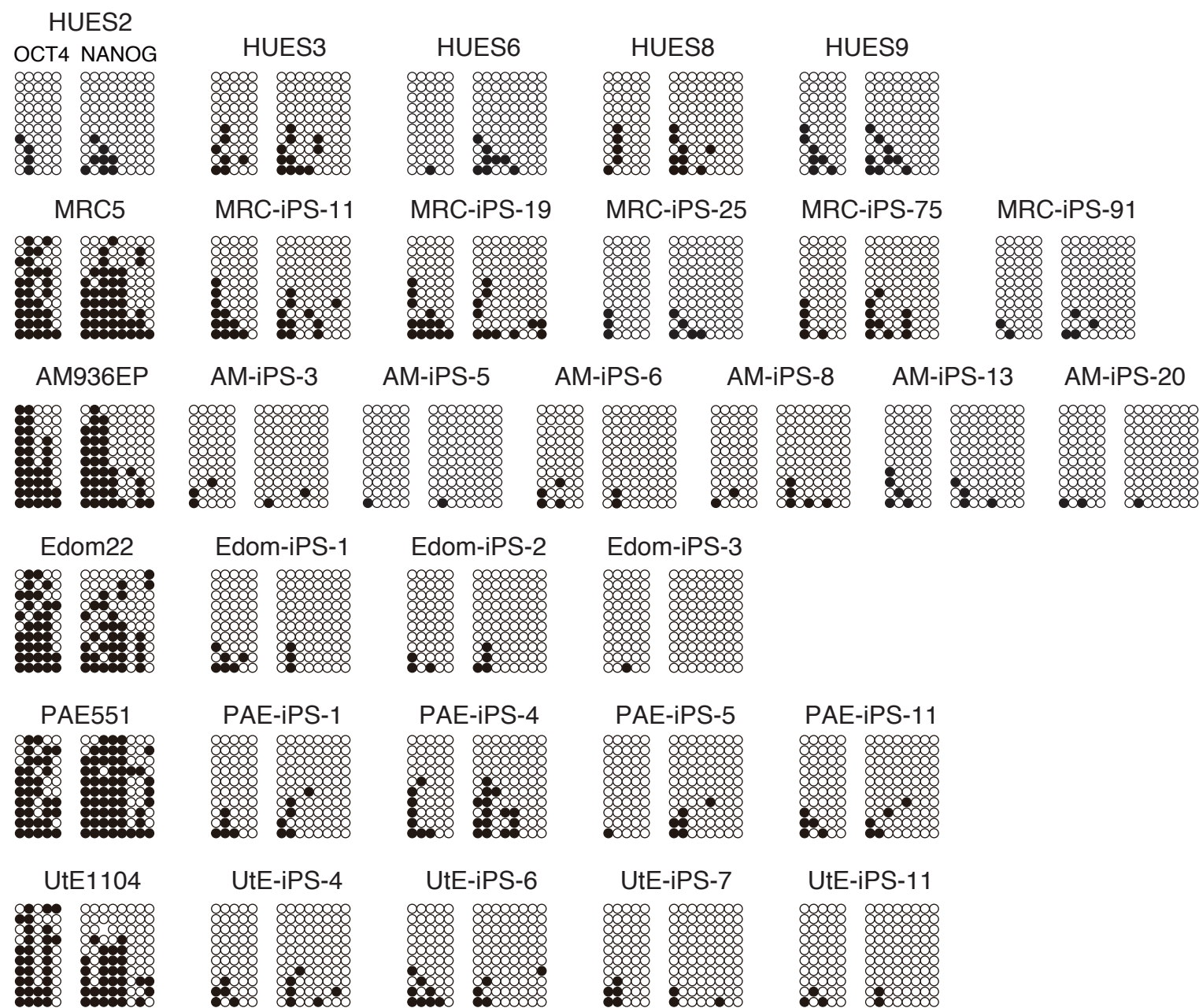

Supplement: Figure S3 — Bisulfite sequencing at the OCT3/4 and NANOG promoter regions in ESCs, iPSCs and their parent cells. (PDF) [file pgen.1002085.s003.pdf]

Figure S4

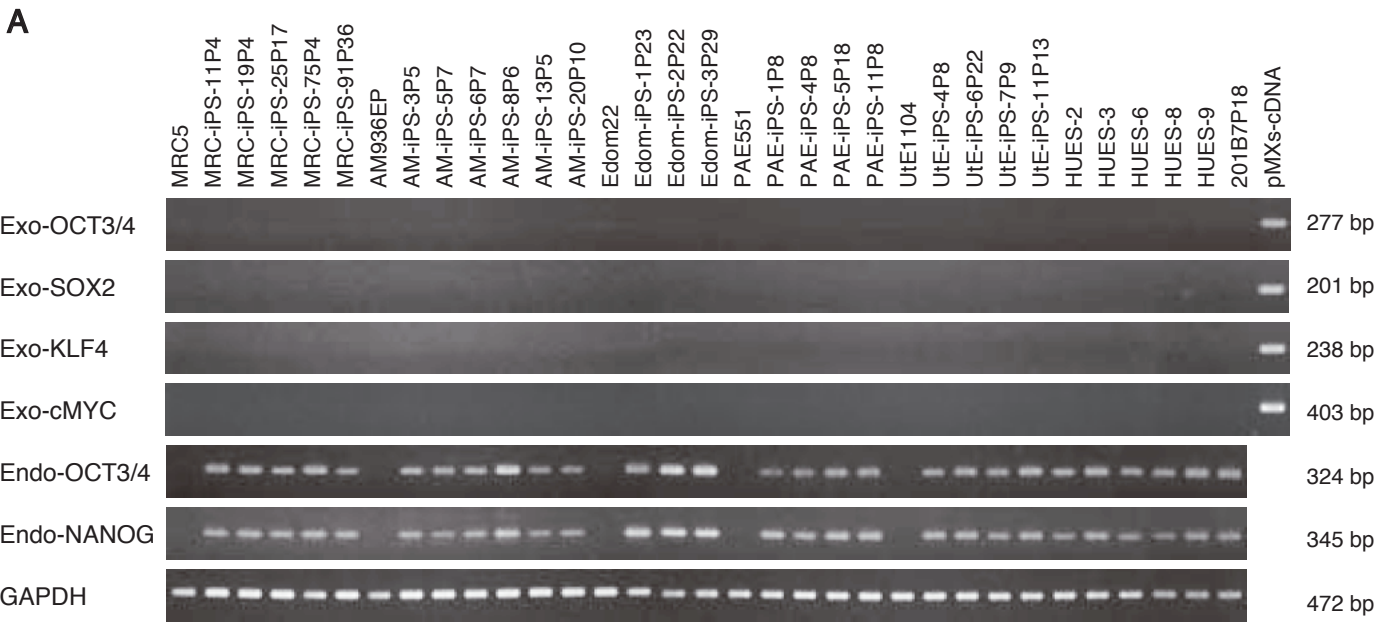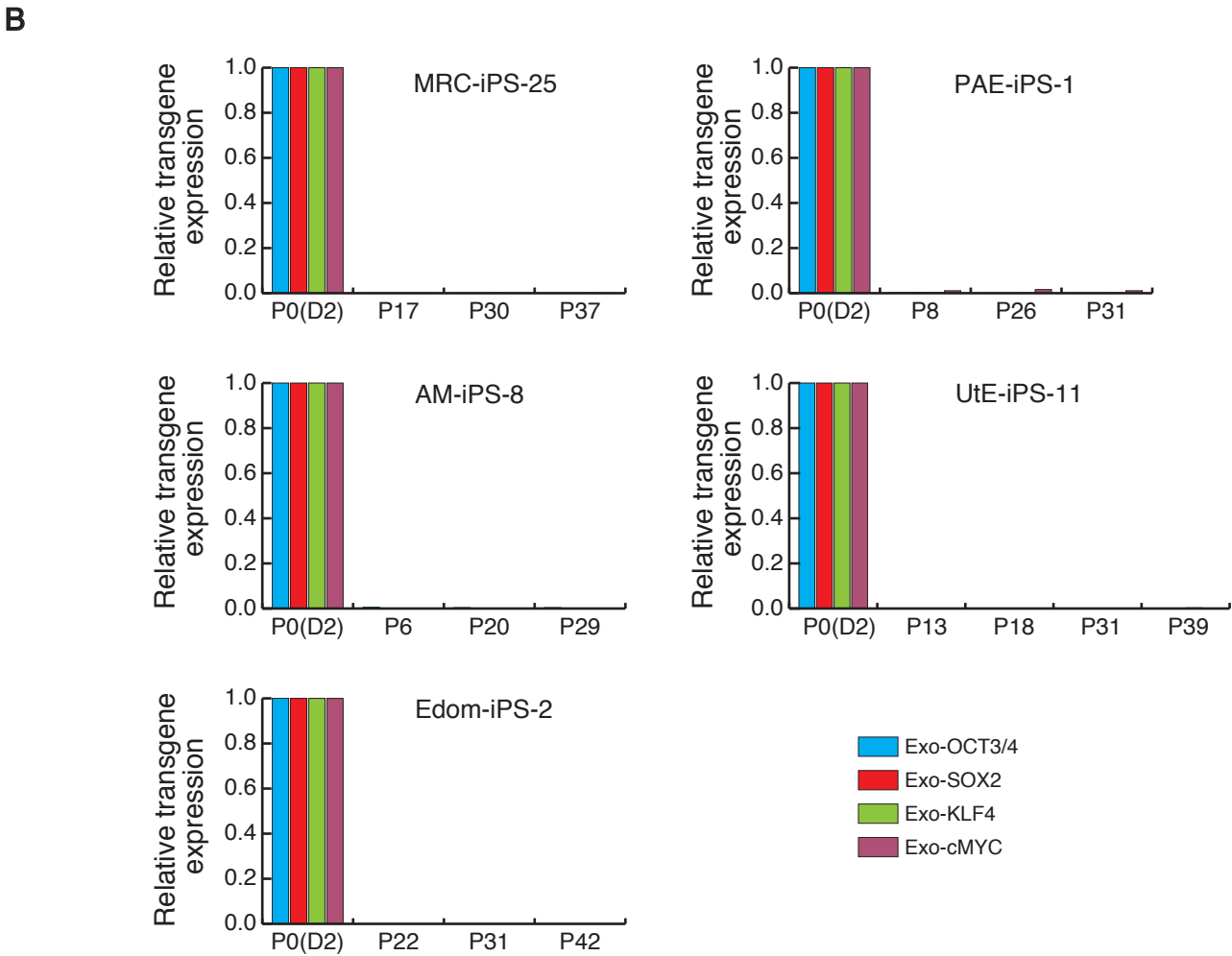

Supplement: Figure S4 — Expression of the transgenes in iPSCs. (A) RT-PCR for transgenes in 22 iPSC lines. No expression of the transgenes in each iPSC lines was detected. (B) Quantitative RT-PCR for the transgenes at each passage. Relative expression of each transgene normalized to GAPDH was calculated. P0(D2), RNA from UtE1104 cells that were infected with the retroviruses and were cultured for 2 days. No expression of the transgenes at each passage was detected. (PDF) [file pgen.1002085.s004.pdf]

Figure S5

A

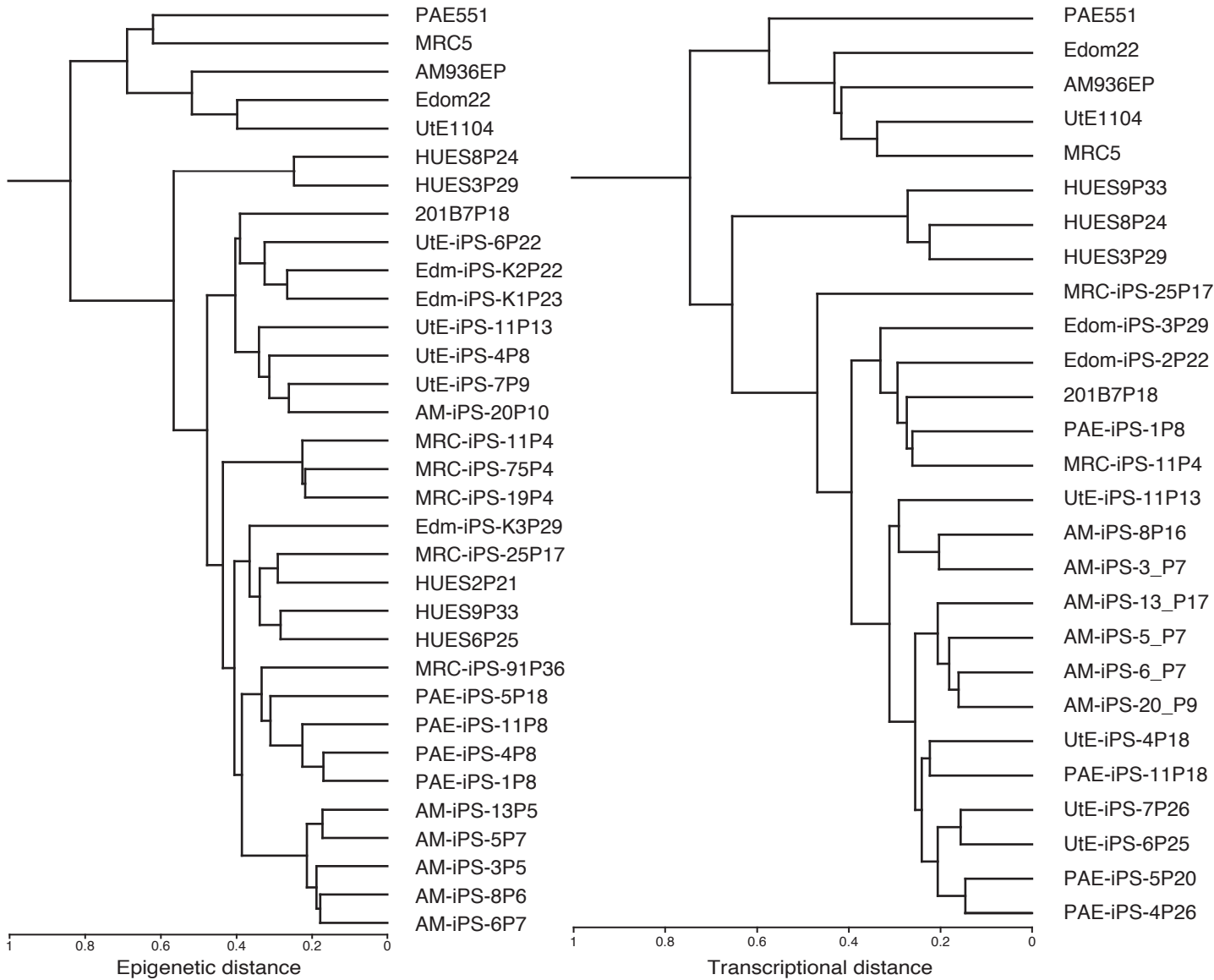

B

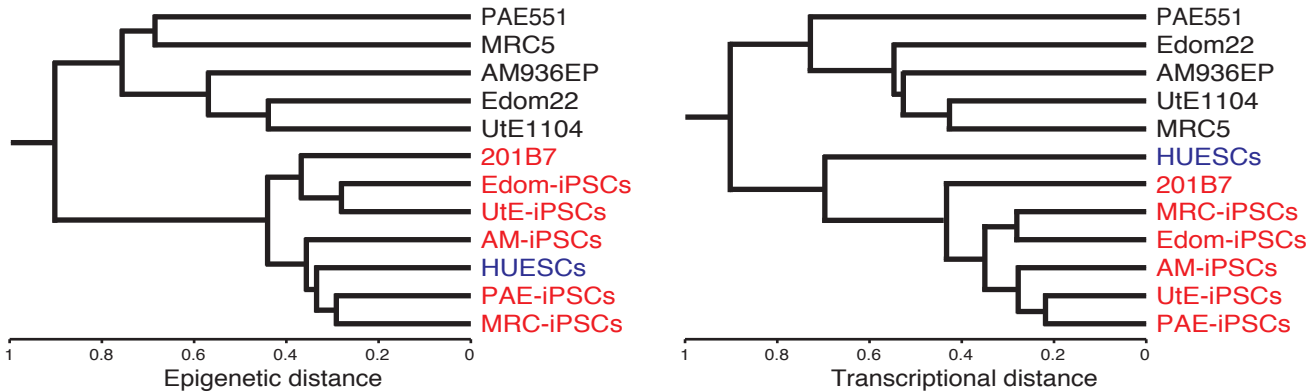

C

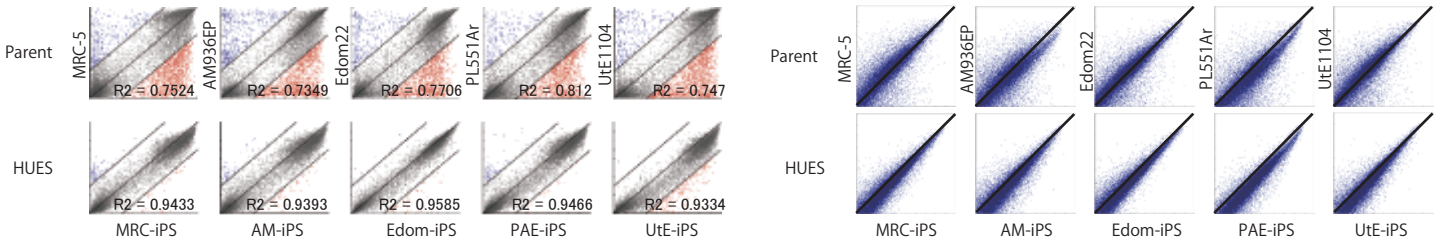

Supplement: Figure S5 — (A) Unsupervised hierarchical clustering analysis based on DNA methylation (left) and gene expression (right) in each ESC line, iPSC line and their parent cell line. (B) Unsupervised hierarchical clustering analysis based on DNA methylation (left) and gene expression (right) of average of ESCs, iPSCs and parent cells. (C) Scatter plot of DNA methylation (left) and gene expression data (right) in ESCs, iPSCs and their parent cells. (PDF) [file pgen.1002085.s005.pdf]

Figure S7

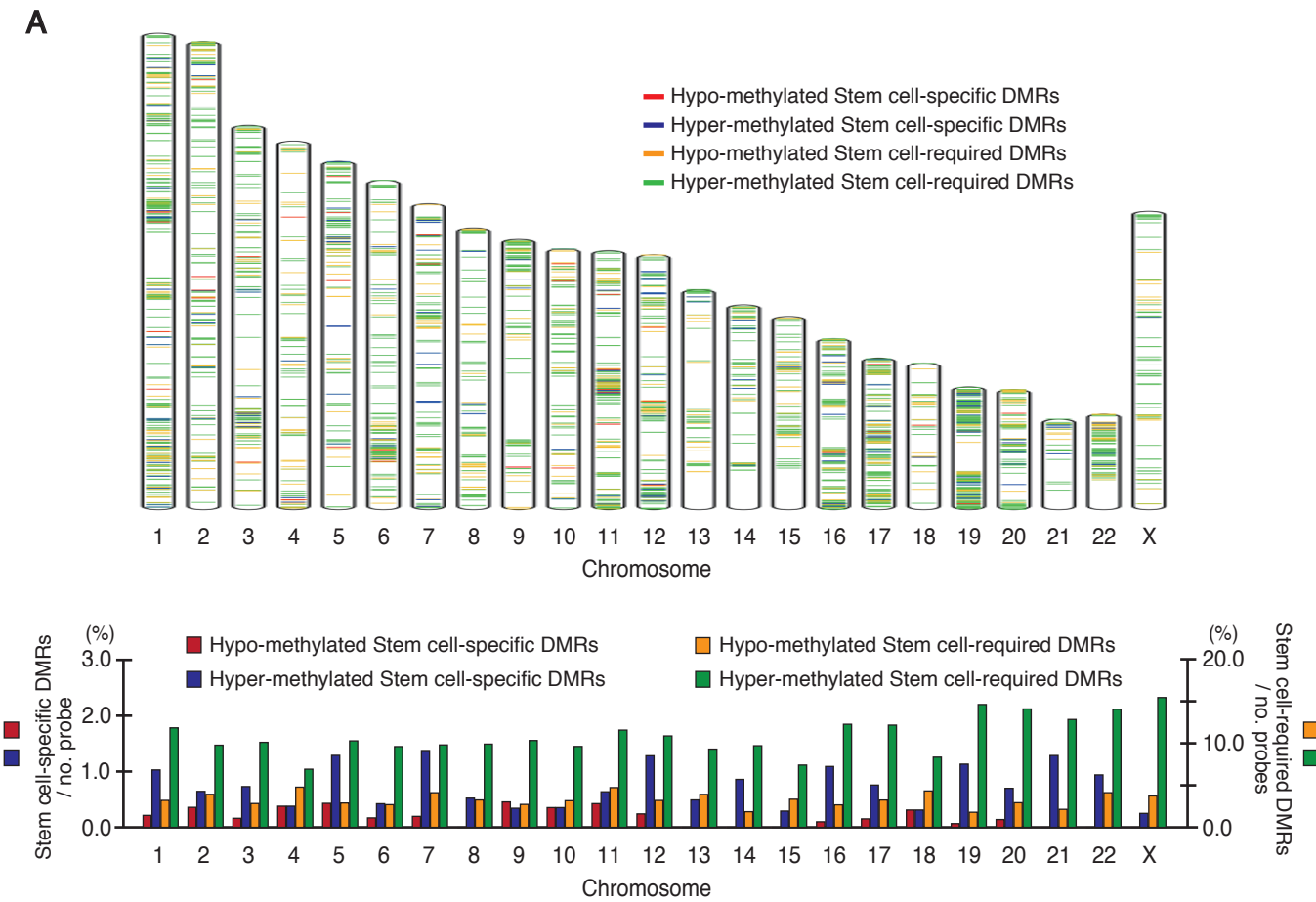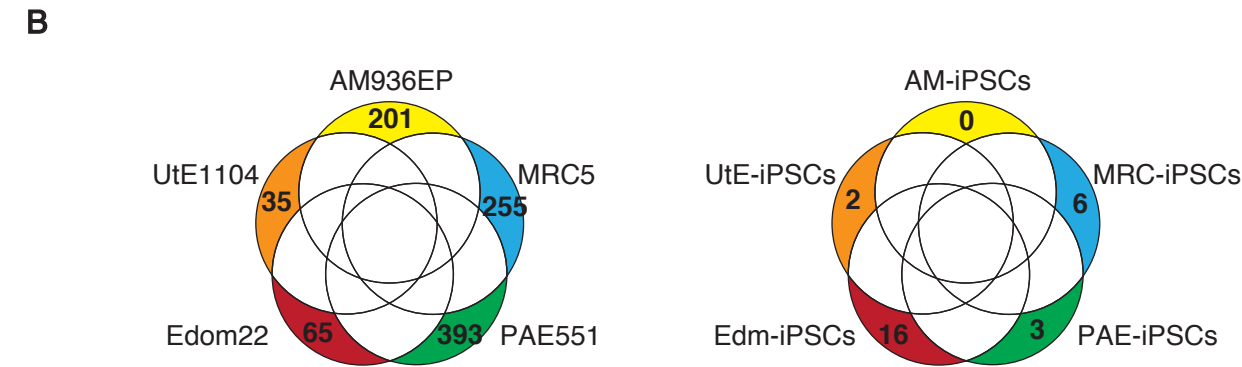

Supplement: Figure S7 — (A) Distribution of stem cell-required DMRs on each chromosome (upper) and frequency on each chromosome (bottom). (B) The number of parent cell specific DMRs (left) and the number of iPSC derived from different parent cells specific DMRs (left). (PDF) [file pgen.1002085.s007.pdf]

Figure S8

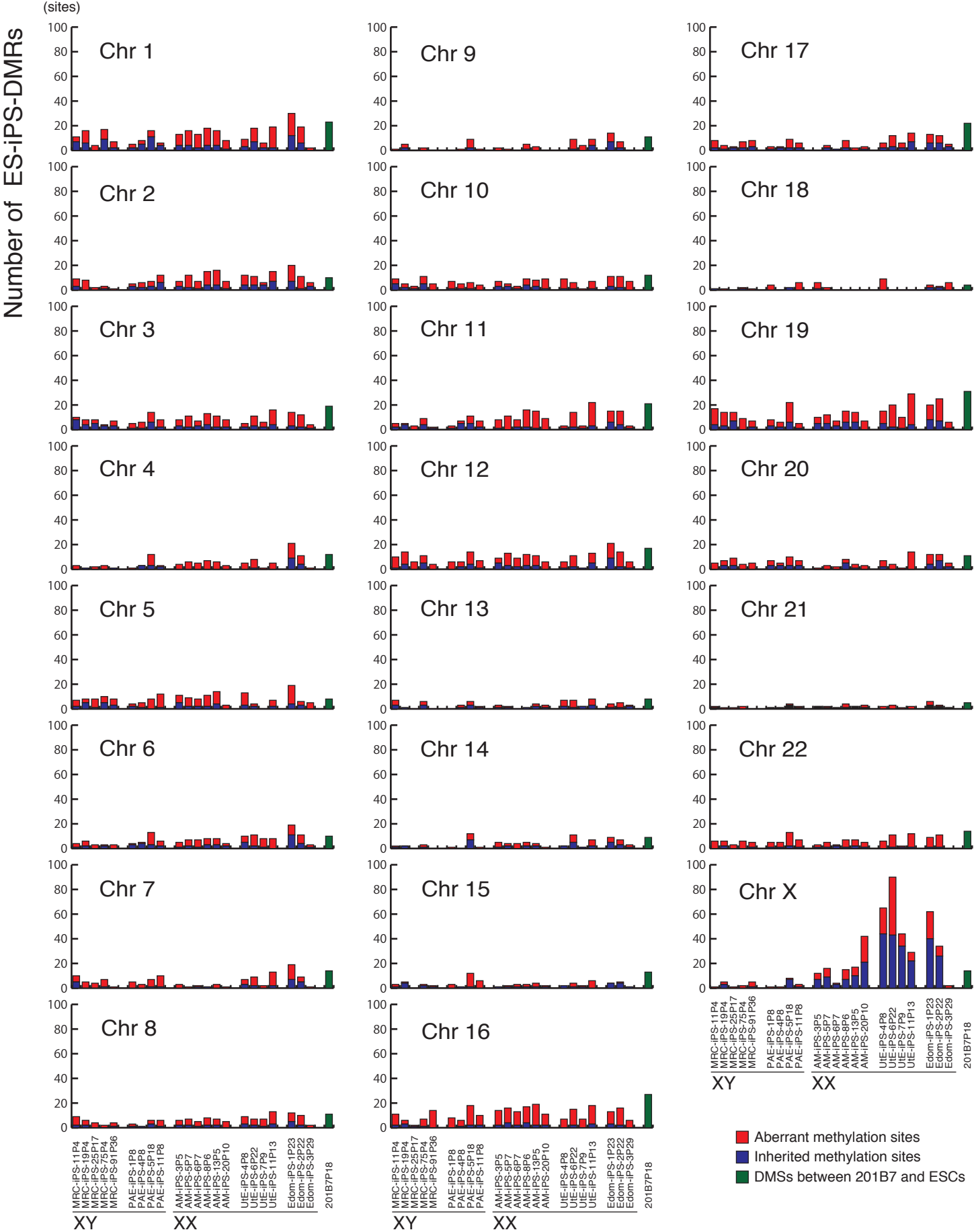

Supplement: Figure S8 — The number of DMRs between ESCs and each iPSC line (ES-iPS-DMRs) on each chromosome. ES-iPS-DMRs between 201B7 (iPSCs from Yamanaka) and ESCs are shown for comparison. (PDF) [file pgen.1002085.s008.pdf]

Figure S9

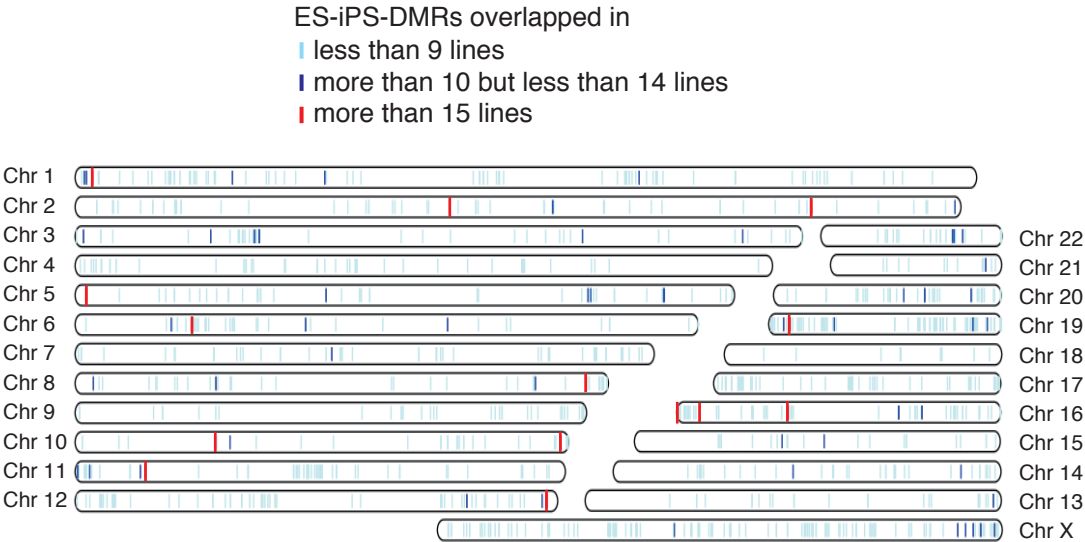

Supplement: Figure S9 — Distribution of the ES-iPS-DMRs on each chromosome. Distribution of the EiP-DMRs overlapped in less than 9 lines (light blue bars), in more than 10 and less than 14 lines (blue bars), and in more than 15 lines (red bars) among 22 lines. (PDF) [file pgen.1002085.s009.pdf]

Figure S10

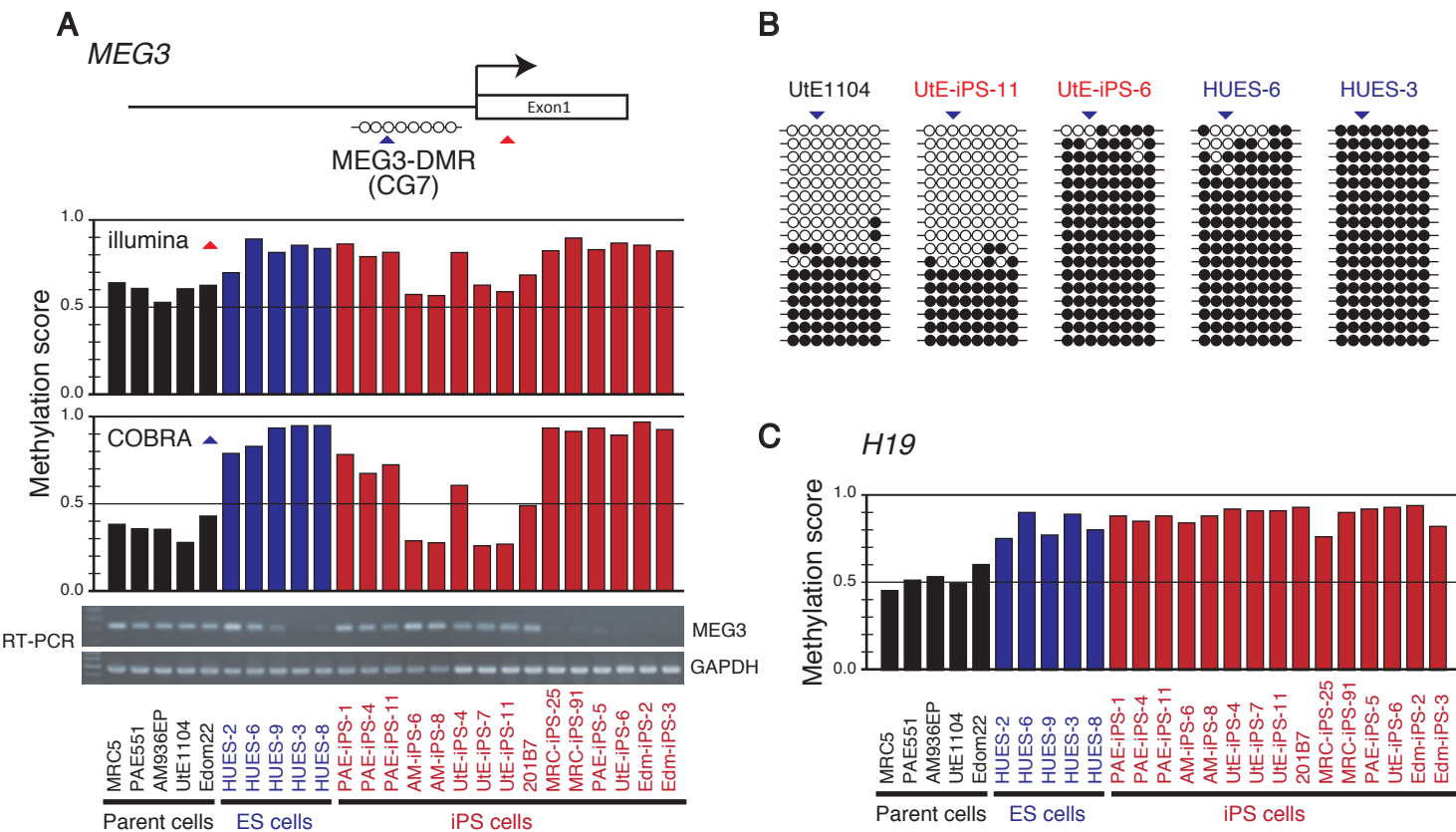

Supplement: Figure S10 — DNA methylation at human MEG3 and H19. (A) DNA methylation at MEG3-DMR (CG7) and expression of MEG3. (Top) Schematic diagram of the MEG3 gene. The arrow, open boxes and open circles represent transcription start site, first exon and position of CpG sites, respectively. Red and blue arrowheads represent the position of CpG sites in Infinium assay and COBRA assay, respectively. DNA methylation scores of MEG3 were determined by Illumina Infinium HumanMethylation27 assay (upper bar graph) and Bio-COBRA (lower bar graph). (Bottom) Expression of MEG3 and GAPDH was determined by RT-PCR. Information of MEG3 primers for COBRA and RT-PCR is described by Kagami et al. [40]. (B) Bisulfite sequencing analysis of MEG3-DMRs (CG7). (C) Methylation scores of H19 were determined by Illumina Infinium HumanMethylation27 assay. (PDF) [file pgen.1002085.s010.pdf]
