# Supplementary material for: DNA Methylation Dynamics in Human Induced Pluripotent Stem Cells over Time
Source: PLoS Genet. 2011 May 26;7(5):e1002085. doi: 10.1371/journal.pgen.1002085 (PMC3102737; doi:10.1371/journal.pgen.1002085)
Supplement: Figure S6 — (A) Venn-like diagram showing seven categories (aa-gg) overlapped CpG sites among ESCs, iPSCs and their parent cells. (B) Number of CpG sites involved in each seven category from the five ESCs-iPSCs-the parent cell sets. “Overlapped” indicates a number of sites that overlap in all iPSCs examined. The 220 overlapping sites in “ee” are designated as stem cell-specific differentially methylated regions (DMRs) and 3,123 total sites in “ee” are designated as stem cell-required DMRs. Notably, no overlapping sites were observed in “bb” that is a category involved in iPSCs-specific DMRs and in “ff” that is a category involved in inherited regions in iPSCs from the parent cells. (PDF) [file pgen.1002085.s006.pdf]

Figure S6

A

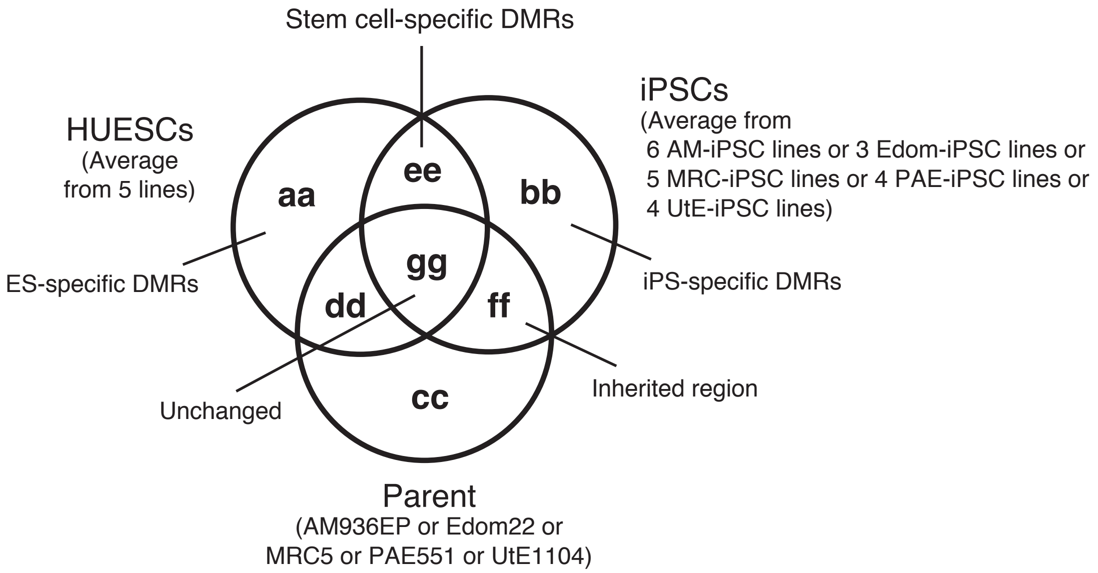

B

Category \ Set	AM-iPSCs AM936EP HUESCs	Edom-iPSCs Edom22 HUESCs	MRC-iPSCs MRC5 HUESCs	PAE-iPSCs PAE551 HUESCs	UtE-iPSCs UtE1104 HUESCs	Overlapped	Total
aa	25	20	6	18	23	0	75
bb	70	38	16	27	46	0	141
cc	1678	1310	1420	1083	1382	227	3125
dd	62	27	12	22	34	0	113
ee	1670	1299	1416	1078	1370	220	3123
ff	17	10	2	13	11	0	43
gg	19717	20074	19931	20502	19898	17572	21713
