# Supplementary material for: DNA Methylation Dynamics in Human Induced Pluripotent Stem Cells over Time
Source: PLoS Genet. 2011 May 26;7(5):e1002085. doi: 10.1371/journal.pgen.1002085 (PMC3102737; doi:10.1371/journal.pgen.1002085)
Supplement: Table S1 — List of human cells analyzed for a methylation state in this study. (PDF) [file pgen.1002085.s011.pdf]

Table S1. List of human cells analyzed for a methylation state in this study.

Cell ID	Description	Karyotype
MRC5	Fetal lung fibroblast cells	46 XY
MRC-iPS-11	MRC5-derived iPS cells (P4)	46 XY
MRC-iPS-19	MRC5-derived iPS cells (P4)	46 XY
MRC-iPS-25	MRC5-derived iPS cells (P17, P30, P37)	46 XY
MRC-iPS-75	MRC5-derived iPS cells (P4)	46 XY
MRC-iPS-91	MRC5-derived iPS cells (P36)	46 XY
AM936EP	Amnion-derived cells (P6)	46 XX
AM-iPS-3	AM936EP-derived iPS cells (P5)	46 XX
AM-iPS-5	AM936EP-derived iPS cells (P7)	46 XX
AM-iPS-6	AM936EP-derived iPS cells (P7)	46 XX
AM-iPS-8	AM936EP-derived iPS cells (P6, P20, P29)	46 XX
AM-iPS-13	AM936EP-derived iPS cells (P5)	46 XX
AM-iPS-20	AM936EP-derived iPS cells (P10)	46 XX
Edom22	Menstrual blood-derived cells (P5, P11, P16)	46 XX
Edom-iPS-1	Edom22-derived iPS cells (P23)	46 XX
Edom-iPS-2	Edom22-derived iPS cells (P22, P31, P42)	46 XX
Edom-iPS-3	Edom22-derived iPS cells (P29)	46 XX
PAE551	Placental artery endothelial cells (P13)	46 XY
PAE-iPS-1	PAE551-derived iPS cells (P8, P26, P31)	46 XY
PAE-iPS-4	PAE551-derived iPS cells (P8)	46 XY
PAE-iPS-5	PAE551-derived iPS cells (P18, P31, P34)	46 XY
PAE-iPS-11	PAE551-derived iPS cells (P8)	46 XY
UtE1104	Endometrium-derived cells (P7, P11, P16)	46 XX
UtE-iPS-4	UtE1104-derived iPS cells (P8 P30)	46 XX
UtE-iPS-6	UtE1104-derived iPS cells (P22)	46 XX
UtE-iPS-7	UtE1104-derived iPS cells (P9)	46 XX
UtE-iPS-11	UtE1104-derived iPS cells (P13, P18, P31, P39)	46 XX
HUES2	Embryonic stem cells (P21)	46 XY
HUES3	Embryonic stem cells (P29)	46 XY
HUES6	Embryonic stem cells (P25)	46 XX
HUES8	Embryonic stem cells (P24)	46 XY
HUES9	Embryonic stem cells (P33)	46 XX
201B7	Fibroblast-derived iPS cells (P18)	46 XX

Numbers in parenthesis with P indicate passage in culture on the cells used in the methylation analysis. HUESCs [23, 24] were kindly gifted from Drs. C. Cowan and T. Tenzan. 201B7 human iPSC line [8] was generated from human skin fibroblasts by retroviral transfection with 4 transcription factors.
