# Supplementary material for: DNA Methylation Dynamics in Human Induced Pluripotent Stem Cells over Time
Source: PLoS Genet. 2011 May 26;7(5):e1002085. doi: 10.1371/journal.pgen.1002085 (PMC3102737; doi:10.1371/journal.pgen.1002085)
Supplement: Table S2 — STR analysis of iPSCs. (PDF) [file pgen.1002085.s012.pdf]

Table S2. STR analysis of iPSCs

Locus	HUES2		HUES3		HUES6		HUES8		HUES9		MRC5		MRC-iPS-11		MRC-iPS-19		MRC-iPS-25		MRC-iPS-75		MRC-iPS-91	
D3S1358	14	18	15	16	16	18	15	19	17	18	15	17	15	17	15	17	15	17	15	17	15	17
TH01	5	6	7	8	8	9.3	7	9	8	9	8		8		8		8		8		8	
D21S11	31	32	28	29	30		28	29	29	30	31.2		31.2		31.2		31.2		31.2		31.2	
D18S51	12	16	13	17	12	14	12	16	12	13	15	21	15	21	15	21	15	21	15	21	15	21
Penta_E	7	15	11	12	12		7	10	10	12	12	16	12	16	12	16	12	16	12	16	12	16
D5S818	11	12	12		12		11		12		11	12	11	12	11	12	11	12	11	12	11	12
D13S317	11	12	8	14	11		12	14	11	12	11	14	11	14	11	14	11	14	11	14	11	14
D7S820	9	13	8	11	8	11	11	12	8	12	10	11	10	11	10	11	10	11	10	11	10	11
D16S539	11	13	12	14	10	11	9	12	12	13	9	11	9	11	9	11	9	11	9	11	9	11
CSF1PO	12		10	12	11	12	10	12	10		11	12	11	12	11	12	11	12	11	12	11	12
Penta_D	11	13	9	12	9	10	10	11	9	13	12		12		12		12		12		12	
AMEL	X	Y	X	Y	X		X	Y	X		X	Y	X	Y	X	Y	X	Y	X	Y	X	Y
vWA	14	16	16	17	15	16	15	19	17	19	15		15		15		15		15		15	
D8S1179	8	12	12	13	10	13	13		9	12	13		13		13		13		13		13	
TPOX	9	11	8	8	9	11	8	11	8	9	8		8		8		8		8		8	
FGA	18	23	22	24	23	24	20	25	19	21	21	23	21	23	21	23	21	23	21	23	21	23

Locus	AM936EP		AM-iPS-3		AM-iPS-5		AM-iPS-6		AM-iPS-8		AM-iPS-13		AM-iPS-20		Edom22		Edom-iPS-1		Edom-iPS-2		Edom-iPS-3	
D3S1358	15	16	15	16	15	16	15	16	15	16	15	16	15	16	15	18	15	18	15	18	15	18
TH01	6	7	6	7	6	7	6	7	6	7	6	7	6	7	7	9	7	9	7	9	7	9
D21S11	29	30	29	30	29	30	29	30	29	30	29	30	29	30	30	31	30	31	30	31	30	31
D18S51	12	19	12	19	12	19	12	19	12	19	12	19	12	19	16		16		16		16	
Penta_E	16		16		16		16		16		16		16		8	18	8	18	8	18	8	18
D5S818	11	13	11	13	11	13	11	13	11	13	11	13	11	13	11		11		11		11	
D13S317	11	13	11	13	11	13	11	13	11	13	11	13	11	13	8	12	8	12	8	12	8	12
D7S820	10	13	10	13	10	13	10	13	10	13	10	13	10	13	9	12	9	12	9	12	9	12
D16S539	13		13		13		13		13		13		13		10	12	10	12	10	12	10	12
CSF1PO	12		12		12		12		12		12		12		12		12		12		12	
Penta_D	9	13	9	13	9	13	9	13	9	13	9	13	9	13	9	11	9	11	9	11	9	11
AMEL	X		X		X		X		X		X		X		X		X		X		X	
vWA	14	19	14	19	14	19	14	19	14	19	14	19	14	19	14	17	14	17	14	17	14	17
D8S1179	13	15	13	15	13	15	13	15	13	15	13	15	13	15	13	14	13	14	13	14	13	14
TPOX	8	9	8	9	8	9	8	9	8	9	8	9	8	9	11		11		11		11	
FGA	21	23	21	23	21	23	21	23	21	23	21	23	21	23	19	24	19	24	19	24	19	24

Locus	PAE551		PAE-iPS-1		PAE-iPS-4		PAE-iPS-5		PAE-iPS-11		UtE1104		UtE-iPS-4		UtE-iPS-6		UtE-iPS-7		UtE-iPS-11	
D3S1358	16	17	16	17	16	17	16	17	16	17	15		15		15		15		15	
TH01	6	9.3	6	9.3	6	9.3	6	9.3	6	9.3	9		9		9		9		9	
D21S11	29	31	29	31	29	31	29	31	29	31	31.2	32.2	31.2	32.2	31.2	32.2	31.2	32.2	31.2	32.2
D18S51	14		14		14		14		14		14		14		14		14		14	
Penta_E	5	11	5	11	5	11	5	11	5	11	5		5		5		5		5	
D5S818	11	13	11	13	11	13	11	13	11	13	12	13	12	13	12	13	12	13	12	13
D13S317	8	12	8	12	8	12	8	12	8	12	8	9	8	9	8	9	8	9	8	9
D7S820	8	11	8	11	8	11	8	11	8	11	8	11	8	11	8	11	8	11	8	11
D16S539	10	13	10	13	10	13	10	13	10	13	10	11	10	11	10	11	10	11	10	11
CSF1PO	10	11	10	11	10	11	10	11	10	11	12		12		12		12		12	
Penta_D	9		9		9		9		9		9	10	9	10	9	10	9	10	9	10
AMEL	X	Y	X	Y	X	Y	X	Y	X	Y	X		X		X		X		X	
vWA	17	18	17	18	17	18	17	18	17	18	16	18	16	18	16	18	16	18	16	18
D8S1179	13	16	13	16	13	16	13	16	13	16	13	16	13	16	13	16	13	16	13	16
TPOX	8		8		8		8		8		8	11	8	11	8	11	8	11	8	11
FGA	22	25	22	25	22	25	22	25	22	25	19	23	19	23	19	23	19	23	19	23
