# Supplementary material for: DNA Methylation Dynamics in Human Induced Pluripotent Stem Cells over Time
Source: PLoS Genet. 2011 May 26;7(5):e1002085. doi: 10.1371/journal.pgen.1002085 (PMC3102737; doi:10.1371/journal.pgen.1002085)
Supplement: Table S3 — Karyotypic analysis of iPSCs. (PDF) [file pgen.1002085.s013.pdf]

Table S3. Karyotypic analysis of iPSCs

Cell ID	Passage	Karyotype	Cell number
MRC5		46,XY	17
		46,XY,+8	1
		46,XY,+10	1
		92,XXYY	1
MRC-iPS-25	10	46,XY	20
	43	46,XY	20
MRC-iPS-91	45	46,XY	20
AM-iPS-3	8	46,XX	20
	43	46,XX	20
	63	46,XX	20
AM-iPS-5	7	46,XX	20
	53	46,XX	20
AM-iPS-6	7	46,XX	20
		46,XX,−3,+1mar	1
		46,XX,add(8)(p23)	1
	53	46,XX	20
AM-iPS-8	18	46,XX	20
AM-iPS-13	9	46,XX	19
		44,XX,−5,−11,−14,−18,+2mar	1
AM-iPS-20	10	46,XX	19
		92,XXXX	1
Edom22	5	46,XX	20
Edom-iPS-1	22	46,XX	20
Edom-iPS-2	21	46,XX	20
Edom-iPS-3	20	46,XX	20
PAE551	13	46,XY	20
PAE-iPS-1	9	46,XY	18
		46,XY,inv(10)(p11.2q22)	1
		46,XY,t(1;6)(p36.1;q25)	1
PAE-iPS-4	9	46,XY	20
PAE-iPS-5	7	46,XY	20
PAE-iPS-11	9	46,XY	19
		45,XY,t(7;11)(q32;q25),−16,−20,+mar	1
	30	46,XY	20
UtE1104	8	46,XX	12
		46,XX,del(4q)	1
UtE-iPS-4	16	46,XX	20
	33	46,XX	20
UtE-iPS-6	10	46,XX	20
UtE-iPS-7	9	46,XX	19
		46,XX,del(9)(q11)	1
UtE-iPS-11	9	46,XX	20
	30	46,XX	20
