# Supplementary material for: DNA Methylation Dynamics in Human Induced Pluripotent Stem Cells over Time
Source: PLoS Genet. 2011 May 26;7(5):e1002085. doi: 10.1371/journal.pgen.1002085 (PMC3102737; doi:10.1371/journal.pgen.1002085)
Supplement: Table S4 — List of genes with stem cell-specific DMRs exhibiting significant changes in expression in human iPS cells. (PDF) [file pgen.1002085.s014.pdf]

Table S4. List of genes with stem cell-specific DMRs exhibiting significant changes in expression in human iPS cells.

TargetID	Gene name	Fold change of expression	DNA methylation level iPS cells	Parental cells
**SS-hypo-DMRs**				
cg07337598	ANXA9, annexin A9	31.5	0.305	0.710
cg17349199	C10orf82, hypothetical protein LOC143379	72.5	0.087	0.684
cg18997129	EPHA1, ephrin receptor EphA1	387.8	0.087	0.700
cg21129531	LRRC4, netrin-G1 ligand	22.4	0.050	0.724
cg24625388	NEBL, nebulette non-muscle isoform	67.8	0.108	0.794
cg04956511	PTPN6, protein tyrosine phosphatase; non-receptor type 6 isoform 1	23.3	0.083	0.920
cg19580810	RAB25	165.8	0.047	0.741
cg06303238	SALL4, sal-like 4	885.1	0.026	0.764
cg03453449	USP44, ubiquitin specific protease 44	111.6	0.042	0.755
**SS-hyper-DMRs**				
cg25193278	BTN3A3, butyrophilin; subfamily 3; member A3 isoform a	0.060	0.721	0.113
cg11375102	C16orf30, claudin-like protein 24	0.005	0.756	0.073
cg13802966	CASP1, caspase 1 isoform delta	0.004	0.869	0.408
cg03714916	CDKN1A, cyclin-dependent kinase inhibitor 1A	0.087	0.730	0.096
cg14409083	EMP1, epithelial membrane protein 1	0.008	0.854	0.055
cg24910675	ENG, endoglin precursor	0.013	0.624	0.047
cg11808544	FKBP9L, FK506 binding protein 9-like	0.111	0.836	0.094
cg13406950	GBP1, guanylate binding protein 1; interferon-inducible; 67kD	0.006	0.815	0.301
cg22074858	GBP3, guanylate binding protein 3	0.004	0.766	0.079
cg12167564	LYST, lysosomal trafficking regulator isoform 1	0.012	0.762	0.060
cg14209518	NNMT, nicotinamide N-methyltransferase	0.003	0.727	0.170
cg18771300	RHOJ, TC10-like Rho GTPase	0.042	0.610	0.059
cg23539753	SP100, nuclear antigen Sp100	0.034	0.763	0.054
cg18727700	SRPX2, sushi-repeat-containing protein; X-linked 2	0.002	0.801	0.105
cg20935106	TAPBPL, TAP binding protein-like	0.185	0.628	0.081
cg05360220	TNFRSF14, tumor necrosis factor receptor superfamily; member 14 precursor	0.015	0.774	0.154
cg16970828	UBE1L, ubiquitin-activating enzyme E1-like	0.026	0.679	0.041

Fold change of expression: Fold change of expression of the listed genes in human iPS cells was estimated by comparing with expression level in parent cells.
