# Supplementary material for: DNA Methylation Dynamics in Human Induced Pluripotent Stem Cells over Time
Source: PLoS Genet. 2011 May 26;7(5):e1002085. doi: 10.1371/journal.pgen.1002085 (PMC3102737; doi:10.1371/journal.pgen.1002085)
Supplement: Table S5 — List of the top 100 genes with hypo-methylated stem cell-required DMRs exhibiting ‘high’ expression in human iPS cells. (PDF) [file pgen.1002085.s015.pdf]

Table S5. List of top 100 genes with hypo-methylated stem cell-required DMRs exhibiting 'high' expression in human iPS cells.

Gene name	
CSNK1E	casein kinase 1 epsilon
GAL	galanin preproprotein
POU5F1	POU domain; class 5; transcription factor 1 isoform 1
IFITM1	interferon induced transmembrane protein 1 (9-27)
F11R	F11 receptor isoform a precursor
SRRM2	splicing coactivator subunit SRm300
TACSTD1	tumor-associated calcium signal transducer 1 precursor
SALL4	sal-like 4
BAMBI	BMP and activin membrane-bound inhibitor precursor
COL9A3	alpha 3 type IX collagen
DDR1	discoidin domain receptor family; member 1 isoform b
LARP1	la related protein isoform 2
CDH1	cadherin 1; type 1 preproprotein
TUBB2B	tubulin; beta polypeptide paralog
CACYBP	calcyclin binding protein isoform 1
C14orf115	hypothetical protein LOC55237
TM7SF2	transmembrane 7 superfamily member 2
KRT8	keratin 8
SLC39A14	solute carrier family 39 (zinc transporter); member 14
ATIC	5-aminoimidazole-4-carboxamide ribonucleotide formyltransferase/IMP cyclohydrolase
SNX2	sorting nexin 2
MLH1	MutL protein homolog 1
VAMP8	vesicle-associated membrane protein 8
SLC7A5	solute carrier family 7 (cationic amino acid transporter; y+ system); member 5
MAD2L2	MAD2 homolog
LEFTY1	left-right determination; factor B preproprotein
RYBP	RING1 and YY1 binding protein
ABHD9	abhydrolase domain containing 9
VWCE	hypothetical protein LOC220001
GPR125	G protein-coupled receptor 125
FBXO2	F-box only protein 2
ETS2	v-ets erythroblastosis virus E26 oncogene homolog 2
NES	nestin
PPP1CC	protein phosphatase 1; catalytic subunit; gamma isoform
NUP50	nucleoporin 50kDa isoform a
ANAPC4	anaphase-promoting complex subunit 4
FGFR1	fibroblast growth factor receptor 1 isoform 1 precursor
USP11	ubiquitin specific protease 11
TSPAN6	transmembrane 4 superfamily member 6
MORF4L1	MORF-related gene 15 isoform 2
FBLN1	fibulin 1 isoform C precursor
C10orf125	hypothetical protein LOC282969
RBM35B	hypothetical protein LOC80004
MEST	mesoderm specific transcript isoform b
ZNF593	zinc finger protein LOC51042
MYCN	v-myc myelocytomatosis viral related oncogene; neuroblastoma derived
PSEN2	presenilin 2 isoform 1
SLC29A1	solute carrier family 29 (nucleoside t ransporters); member 1
FGFR2	fibroblast growth factor receptor 2 isoform 1 precursor

Gene name	
CARD10	caspase recruitment domain protein 10
CITED4	Cbp/p300-interacting transactivator; with Glu/Asp-rich carboxy-terminal domain; 4
DNMT3A	DNA cytosine methyltransferase 3 alpha isoform a
FAM46B	hypothetical protein LOC115572
SLC16A5	solute carrier family 16; member 5
EFS	embryonal Fyn-associated substrate isoform 2
KRTCAP3	keratinocyte associated protein 3
USP10	ubiquitin specific protease 10
NUDT4	nudix-type motif 4 isoform alpha
CRABP2	cellular retinoic acid binding protein 2
COL1A2	alpha 2 type I collagen
TNFRSF25	tumor necrosis factor receptor superfamily; member 25 isoform 2 precursor
CXCL12	chemokine (C-X-C motif) ligand 12 (stromal cell-derived factor 1) isoform alpha
RRM2	ribonucleotide reductase M2 polypeptide
ASF1A	ASF1 anti-silencing function 1 homolog A
MLPH	melanophilin
USP44	ubiquitin specific protease 44
PPP1R9A	protein phosphatase 1; regulatory (inhibitor) subunit 9A isoform 1
ISYNA1	myo-inositol 1-phosphate synthase A1
AMT	aminomethyltransferase (glycine cleavage system protein T)
COG2	component of oligomeric golgi complex 2
LRP8	low density lipoprotein receptor-related protein 8 isoform 3 precursor
MID1	midline 1 isoform alpha
DKFZP686A01247	hypothetical protein LOC22998
RBM35A	hypothetical protein LOC54845 isoform 1
ICAM3	intercellular adhesion molecule 3 precursor
KIAA0240	hypothetical protein LOC23506
LOXL3	lysyl oxidase-like 3 precursor
IFRD1	interferon-related developmental regulator 1
SOX8	SRY (sex determining region Y)-box 8
HIST1H2BO	histone H2B
SLMAP	sarcolemma associated protein
PPP1R13L	protein phosphatase 1; regulatory (inhibitor) subunit 13 like
TCF12	transcription factor 12 isoform b
SATB1	special AT-rich sequence binding protein 1
RBP1	retinol binding protein 1; cellular
CHST7	carbohydrate (N-acetylglucosamine 6-O) sulfotransferase 7
SPINT1	hepatocyte growth factor activator inhibitor 1 isoform 1 precursor
SERPINE2	plasminogen activator inhibitor type 1; member 2
EPHA1	ephrin receptor EphA1
NFKBIB	nuclear factor of kappa light polypeptide gene enhancer in B-cells inhibitor; beta isoform a
ACTN3	skeletal muscle specific actinin; alpha 3
SOCS1	suppressor of cytokine signaling 1
PRSS8	prostasin preproprotein
FAM57B	hypothetical protein LOC83723
POMC	proopiomelanocortin
KCNK6	potassium channel; subfamily K; member 6
BSCL2	seipin
OTX2	orthodenticle 2 isoform a
RIMS3	regulating synaptic membrane exocytosis 3
C17orf76	hypothetical protein LOC38834
