# Supplementary material for: DNA Methylation Dynamics in Human Induced Pluripotent Stem Cells over Time
Source: PLoS Genet. 2011 May 26;7(5):e1002085. doi: 10.1371/journal.pgen.1002085 (PMC3102737; doi:10.1371/journal.pgen.1002085)
Supplement: Table S6 — List of top 100 genes with hyper-methylated stem cell-required DMRs exhibiting suppression in human iPS cells. (PDF) [file pgen.1002085.s016.pdf]

Table S6. List of top 100 genes with hyper-methylated stem cell-required DMRs exhibiting suppression in human iPS cells.

Gene name	
CETP	cholesteryl ester transfer protein; plasma precursor
KLHL6	kelch-like 6
EMILIN1	elastin microfibril interfacer 1
EGFL7	EGF-like-domain; multiple 7
RNASE1	pancreatic ribonuclease precursor
ROBO4	roundabout homolog 4; magic roundabout
SH3TC1	SH3 domain and tetratricopeptide repeats 1
EDG6	endothelial differentiation; G protein coupled receptor 6 precursor
PECAM1	platelet/endothelial cell adhesion molecule (CD31 antigen)
GJA12	connexin46.6
EMCN	endomucin
GJA4	connexin 37
C21orf84	hypothetical protein LOC114038
LGALS3BP	galectin 3 binding protein
EMP1	epithelial membrane protein 1
ART4	ADP-ribosyltransferase 4 precursor
BMX	BMX non-receptor tyrosine kinase
C20orf175	hypothetical protein LOC140876
SCARF1	scavenger receptor class F; member 1 isoform 2 precursor
GBP1	guanylate binding protein 1; interferon-inducible; 67kD
LGALS1	beta-galactoside-binding lectin precursor
SIRT2	sirtuin 2 isoform 1
CD248	tumor endothelial marker 1 precursor
CLDN5	claudin 5
LAPTM5	Lysosomal associated multispanning membrane protein 5
PPAPDC3	phosphatidic acid phosphatase type 2 domain containing 3
LGALS9	galectin 9 long isoform
MMRN2	multimerin 2
CLIC3	chloride intracellular channel 3
TNFSF18	tumor necrosis factor (ligand) superfamily; member 18
ABCA4	ATP-binding cassette; sub-family A member 4
PDLIM4	PDZ and LIM domain 4
CASP10	caspase 10 isoform a preproprotein
IFI27	interferon; alpha-inducible protein 27
URP2	UNC-112 related protein 2 short form
VWF	von Willebrand factor precursor
CCRL2	chemokine (C-C motif) receptor-like 2
CPA1	pancreatic carboxypeptidase A1 precursor
STAB1	stabilin 1 precursor
OSBPL5	oxysterol-binding protein-like protein 5 isoform a
LCP2	lymphocyte cytosolic protein 2
SDSL	serine dehydratase-like
GPNMB	glycoprotein (transmembrane) nmb isoform b precursor
TAPBPL	TAP binding protein-like
LPIN1	lipin 1
TMEM58	transmembrane protein 58
PIK4CB	phosphatidylinositol 4-kinase; catalytic; beta polypeptide
FMOD	fibromodulin precursor
PTGES	prostaglandin E synthase
GRAP	GRB2-related adaptor protein
COL6A3	alpha 3 type VI collagen isoform 1 precursor
IL1A	interleukin 1; alpha proprotein

Gene name	
SNCG	synuclein; gamma (breast cancer-specific protein 1)
POLD4	polymerase (DNA-directed); delta 4
SEMA3G	semaphorin sem2
FKBP9L	FK506 binding protein 9-like
LY96	MD-2 protein
TBC1D10A	TBC1 domain family; member 10A
TNFRSF14	tumor necrosis factor receptor superfamily; member 14 precursor
C1orf90	hypothetical protein LOC84734
FAIM3	Fas apoptotic inhibitory molecule 3
GNG12	G-protein gamma-12 subunit
RAC2	ras-related C3 botulinum toxin substrate 2
CARD8	caspase recruitment domain family; member 8
CSF1R	colony stimulating factor 1 receptor precursor
IL17RC	interleukin 17 receptor C isoform 3 precursor
SULT1B1	sulfotransferase family; cytosolic; 1B; member 1
C16orf30	claudin-like protein 24
ABTB1	ankyrin repeat and BTB (POZ) domain containing 1 isoform 1
MSN	moesin
HYAL2	hyaluronoglucosaminidase 2
ACTA2	alpha 2 actin
GCKR	glucokinase regulatory protein
ESM1	endothelial cell-specific molecule 1 precursor
LRRC32	leucine rich repeat containing 32 precursor
ARHGDIB	Rho GDP dissociation inhibitor (GDI) beta
AGT	angiotensinogen preproprotein
EML3	echinoderm microtubule associated protein like 3
APOL6	apolipoprotein L6
ZDHHC1	zinc finger; DHHC domain containing 1
FRK	fyn-related kinase
PPP3CC	protein phosphatase 3 (formerly 2B); catalytic subunit; gamma isoform (calcineurin A gamma)
MR1	major histocompatibility complex; class I-related
CAP2	adenylyl cyclase-associated protein 2
TMEM109	hypothetical protein LOC79073
CALCOCO2	calcium binding and coiled-coil domain 2
BTN3A3	butyrophilin; subfamily 3; member A3 isoform a
TGFB1I1	androgen receptor coactivator ARA55
ADPRHL1	ADP-ribosylhydrolase like 1 isoform 2
HTR2B	5-hydroxytryptamine (serotonin) receptor 2B
TLR1	toll-like receptor 1
CARD9	caspase recruitment domain protein 9
CDKN1A	cyclin-dependent kinase inhibitor 1A
TGM2	transglutaminase 2 isoform a
CUL7	cullin 7
AQP8	aquaporin 8
TM4SF18	transmembrane 4 L six family member 18
DPEP2	dipeptidase 2
PXN	paxillin
CASP1	caspase 1 isoform delta
