# Supplementary material for: DNA Methylation Dynamics in Human Induced Pluripotent Stem Cells over Time
Source: PLoS Genet. 2011 May 26;7(5):e1002085. doi: 10.1371/journal.pgen.1002085 (PMC3102737; doi:10.1371/journal.pgen.1002085)
Supplement: Table S7 — List of top 5 categories of GO Term in “Stem cell-required DMRs”. (PDF) [file pgen.1002085.s017.pdf]

Table S7. List of top 5 categories of GO Term in "Stem cell-required DMRs".

Molecylar Function

PantherID: Go Term	Count. Genes	%
**Genes with hypo-methylated stem cell-requiring DMRs**		
MF00042:Nucleic acid binding	195	36.1%
MF00213:Non-receptor serine /threonine protein kinase	112	20.7%
MF00262:Non-motor actin binding protein	84	15.5%
MF00036:Transcription factor	82	15.2%
MF00101:Guanyl-nucleotide exchange factor	80	14.8%
**Genes with hyper-methylated stem cell-requiring DMRs**		
MF00042:Nucleic acid binding	559	30.8%
MF00213:Non-receptor serine /threonine protein kinase	394	21.7%
MF00262:Non-motor actin binding protein	322	17.7%
MF00212:Other G-protein modulator	281	15.5%
MF00001:Receptor	254	14.0%

Biological Process

PantherID: Go Term	Count. Genes	%
**Genes with SR-hypo-DMRs**		
BP00044:mRNA transcription regulation	250	46.3%
BP00040:mRNA transcription	164	30.4%
BP00071:Proteolysis	131	24.3%
BP00102:Signal transduction	125	23.1%
BP00150:MHCI-mediated immunity	117	21.7%
**Genes with SR-hyper-DMRs**		
BP00044:mRNA transcription regulation	728	40.1%
BP00102:Signal transduction	476	26.2%
BP00040:mRNA transcription	451	24.8%
BP00071:Proteolysis	433	23.9%
BP00143:Cation transport	376	20.7%
