# Supplementary material for: DNA Methylation Dynamics in Human Induced Pluripotent Stem Cells over Time
Source: PLoS Genet. 2011 May 26;7(5):e1002085. doi: 10.1371/journal.pgen.1002085 (PMC3102737; doi:10.1371/journal.pgen.1002085)
Supplement: Table S8 — Primer list. (PDF) [file pgen.1002085.s018.pdf]

Table S8. Primer list.

**Primers for bisulfite PCR**		Annealing temp.
Bis-EPHA1-F	5’-tTtgaatttgattaTTagtTtagT-3’	
Bis-EPHA1-R	5’-ctcccaAAaAcaAattActAAcctA-3’	57˚C
Bis-PTPN6-F	5’-TtgTagtgTTattggTTtggTaggT-3’	
Bis-PTPN6-R	5’-tAAaAaAaacaAatacacacttAtccaA-3’	55˚C
Bis-RAB25-F	5’-aTagattTTagaagTTTagTaatgT-3’	
Bis-RAB25-R	5’-tAaaAacaaaAttataatcttcctcaA-3’	55˚C
Bis-SALL4-F	5’-ggTTaatTagTtgtTagggTtTatga-3’	
Bis-SALL4-R	5’-ccccaAccccactcacccaActcc-3’	57˚C
Bis-GBP3-F	5’-gaaattgTatttatggttaTtgagtT-3’	
Bis-GBP3-R	5’-cctAtAtctcacatcaAActcaActA-3’	55˚C
Bis-LYST-F	5’-TaaagTTaaaaggtTattgggatggT-3’	
Bis-LYST-R	5’-taAttttatAtcctccaaAAactAcaA-3’	55˚C
Bis-SP100-F	5’-gTtgtgtattgTaTaaaTaagtggaT-3’	
Bis-SP100-R	5’-tAAAcctcaAaAcctAAcctctAaAc-3’	57˚C
Bis-UBE1L-F	5’-aTTTtTaggtTtgaTaTtTagagaT-3’	
Bis-UBE1L-R	5’-caAAaccctAActccctAaatcctctA-3’	57˚C

*Upper cases in primer sequences represent converted nucleotide for bisulfite PCR

**Primers for RT-PCR**		Annealing temp.
EPHA1-F	5’-GTCGTCACAAAGCGAAAGC-3’	
EPHA1-R	5’-AGCACCTCCCACATCACAAT-3’	60˚C
PTPN6-F	5’-CGTGCTTTCTGTGCTCAGTG-3’	
PTPN6-R	5’-GCAGCTGGTTCTTGATGTAGTT-3’	60˚C
RAB25-F	5’-CCCTCCTGGTGTTTGACCTA-3’	
RAB25-R	5’-AAGGTCAGAGGCTGATGCAAC-3’	60˚C
SALL4-F	5’-GAAAACGGTTCCGTGGAGAG-3’	
SALL4-R 5	’-ACGAGAAGTTCTTCCCACACC-3’	60˚C
GBP3-F	5’-AACCCCTCACACCAGATGAG-3’	
GBP3-R	5’-CAGATGGTCCACATCCTTGA-3’	60˚C
LYST-F	5’-CTTGTCCATGGTCGAGGATT-3’	
LYST-R	5’-TGGCTTCTGAATCTGAGGTG-3’	60˚C
SP100-F	5’-ACCGAGAAGTGAGCCTGTGA-3’	
SP100-R	5’-GCTGTGATCCTGACCCTCTTC-3’	60˚C
UBE1L-F	5’-GTTGGTAGACCCAAGGCAGAG-3’	
UBE1L-R	5’-TGTCTGTGGCTCATCCATGT-3’	60˚C
OCT3/4-F	5’-CGAGCAATTTGCCAAGCTCCTGAA-3’	
OCT3/4-R	5’-TTCGGGCACTGCAGGAACAAATTC-3’	60˚C
NANOG-F	5’-TCCAGCAGATGCAAGAACTCTCCA-3’	
NANOG-R	5’-TCCAGGCCTGATTGTTCCAGGATT-3’	60˚C
GAPDH-F	5’-GCTCAGACACCATGGGGAAGGT-3’	
GAPDH-R	5’-GTGGTGCAGGAGGCATTGCTGA-3’	60˚C
FY-11	5’- AGTAGACGGCATCGCAGCTTG -3’	
FY-12	5’- GCTAGCTTGCCAAACCTACAG -3’	
OCT3/4-SR	5’- AGCCAGGTCCGAGGATCAAC -3’	
NANOG-SR	5’- GCTTCAGCTCCGTCTCCATC -3’	
KLF4-SF	5’- GGCACTACCGTAAACACACG -3’	
cMYC-SF	5’- ACCGAGGAGAATGTCAAGAG -3’	60˚C
